# Ginkgolic Acids Degradation by the *Ginkgo biloba* Endophytic Fungus *Fusarium* sp. DLT-118

**DOI:** 10.3390/foods15071247

**Published:** 2026-04-06

**Authors:** Lu-Ting Dai, Zhi-Fang Yu, You-Xing Zhao, Yi Zheng

**Affiliations:** 1 School of Food Science and Health, Jiangsu Agri-Animal Husbandry Vocational College, Taizhou 225300, China; dailuting121@163.com; 2 Key Laboratory of Research and Development of Natural Product from Li Folk Medicine of Hainan Province & National Key Laboratory for Tropical Crop Breeding, Institute of Tropical Bioscience and Biotechnology, Chinese Academy of Tropical Agricultural Sciences, Haikou 571101, China; 3 College of Food Science and Technology, Nanjing Agricultural University, Nanjing 210095, China; yuzhifang@njau.edu.cn

**Keywords:** ginkgolic acids, biodegradation, *Ginkgo biloba*, endophytic fungus, *Fusarium* sp.

## Abstract

Ginkgolic acids (GAs), the principal toxic constituents in *Ginkgo biloba*, pose health risks including cytotoxicity, allergenicity, and pro-inflammatory effects, limiting the application of Ginkgo resources in the food and health product industries. Developing efficient and environmentally friendly removal methods is essential. The endophytic fungus *Fusarium* sp. DLT-118, isolated from *Ginkgo biloba*, degraded 96.47% of GAs in *Ginkgo biloba* leaf extract (GE) at an initial concentration of 1 mg/mL within 7 days at 28 °C, while concurrently enhancing the antioxidant activity of GE, as indicated by a reduction in the 2,2-diphenyl-1-picrylhydrazyl (DPPH) radical scavenging IC_50_ from 755.7 μg/mL to 544.6 μg/mL. Morphological and oxidative stress analyses showed critical cellular adaptations and stress responses under degradation conditions. Integrated multi-omics analysis indicated that GE stress induced the remodeling of fungal amino acid, lipid, and energy metabolism, as well as the adjustment of membrane and transport functions, to facilitate GAs detoxification. Cytotoxicity assays indicated no significant cytotoxicity of the degradation products towards human normal lung epithelial cells (Beas-2B) and gastric mucosal epithelial cells (GES-1). These findings highlight *Fusarium* sp. DLT-118 as a promising agent for the efficient removal of GAs, offering a potential strategy for the production of GA-reduced Ginkgo-based food and health products.

## 1. Introduction

*Ginkgo biloba* L., an ancient relict tree species from the late Paleozoic era [1], is often regarded as a “living fossil” due to its evolutionarily conserved morphology [2]. It serves functional roles in edible applications, traditional medicine, and urban greening [3,4]. China is a major contributor to global ginkgo production, providing essential raw material support for related industries [5,6]. The therapeutic potential of *Ginkgo biloba* leaf extract (GE) stems from its rich bioactive composition [7], notably flavonoids and ginkgolides [8,9,10]. Consequently, GE is utilized in functional foods and pharmaceutical formulations aimed at addressing conditions like cardiovascular and cerebrovascular diseases, Alzheimer’s disease, and cancers [11,12,13,14,15,16]. However, the application of GE is limited due to the co-extraction of toxic ginkgolic acids (GAs, e.g., C13:1, C15:0, C15:1, C17:1, and C17:2), whose associated cytotoxicity, genotoxicity, allergenicity, and embryotoxicity restrict its safe dosage and clinical or nutraceutical use [17,18]. Therefore, reducing the content of GAs is essential for the development of food-grade *Ginkgo biloba* ingredients and related dietary supplements, as well as for promoting the sustainable growth of the functional food and nutraceutical industries [19,20].

Currently, the detoxification of toxic compounds in food and agricultural products primarily relies on three categories of methods, namely physical, chemical, and biological approaches. Conventional physical techniques, such as thermal processing [21], adsorption [22], and irradiation [23], are relatively simple to operate but may affect material quality and their effectiveness is often limited by environmental conditions. Chemical methods, such as oxidation, reduction, and acid-base treatments, can disrupt toxin structures but are prone to introducing reagent residues or generating unknown by-products, posing a risk of secondary contamination and requiring rigorous safety assessments [24]. In contrast, biological detoxification which encompasses both microbial and enzymatic strategies have gained prominence due to its environmental compatibility, efficiency, and controllability. Central to this approach is biodegradation, wherein microorganisms or enzymes catalytically break down toxic compounds into less harmful or nontoxic products. These biodegradation-based processes provide multiple advantages for eliminating toxins [25] such as patulin, Alternaria toxins, and zearalenone, including low operational cost, the ability to achieve near-complete mineralization of pollutants thus preventing secondary contamination and generally avoiding the formation of hazardous by-products. Hence, developing detoxification strategies that are safe, environmentally sustainable, efficient, and economically viable remains a vital research direction in this field.

In the context of GAs biodegradation, studies have confirmed that certain bacteria, such as *Lactobacillus* [26,27] and *Pantoea* [28], as well as fungi including *Eurotium cristatum* [29] and *Fusarium* species [28] possess degradation potential, with highly efficient strains achieving degradation rates exceeding 90%. Endophytic fungi colonize the interior of plant tissues and establish long-term, stable symbiotic and co-evolutionary relationships with their host plants [30], thereby constituting an underexplored yet highly promising resource for biodegradation. These fungi have evolved the ability to metabolize a variety of plant secondary metabolites, including toxic and antinutritional compounds [31]. Based on this trait, their application in the detoxification of plant-derived toxins has gradually developed into an efficient and environmentally friendly bioremediation strategy [32,33]. Building on the above background, we propose the research hypothesis that endophytic fungi may serve as efficient and ecologically compatible functional agents for the biological detoxification method of GAs. Systematic exploration of novel endophytic strains holds promise for enhancing the degradation efficiency of GAs. Combined with the metabolic plasticity and detoxification potential acquired through long-term symbiosis, the application prospects of endophytic fungi in this field warrant further investigation.

This study investigated the biodegradation of GAs in GE using the endophytic fungus *Fusarium* sp. DLT-118. The reduction in GAs content was quantitatively measured by tracking the decrease in the characteristic high-performance liquid chromatography (HPLC) peak areas of five major GAs (C13:1, C15:0, C15:1, C17:1, and C17:2). Fungal morphological changes during degradation were examined, and integrated transcriptomic and metabolomic analyses, together with oxidative-stress response assays, were employed to elucidate the underlying biodegradation mechanisms. Cytotoxicity assays confirmed that the fermented extract showed no significant toxicity toward human normal cell lines. These findings establish *Fusarium* sp. DLT-118 as a promising microbial agent for GAs detoxification, offering a sustainable, bio-based strategy for developing safer Ginkgo-derived products.

## 2. Materials and Methods

### 2.1. Main Materials and Reagents

Endophytic fungi were isolated from fresh and healthy *Ginkgo biloba* tissues, including leaves, branches, and trunks, collected from the campus of Jiangsu Agri-animal Husbandry Vocational College in Taizhou, China, in March 2025.

Dried *Ginkgo biloba* leaf powder was provided by Taizhou Xiyang Food Co., Ltd. (Taizhou, Jiangsu, China). The raw leaves were cultivated in Taizhou, harvested in October 2024 from the outer and middle canopy layers to ensure maturity, and stored as powder in a desiccator at room temperature in the dark until use.

The reagents and materials were sourced as follows: total ginkgolic acids (HPLC ≥ 98%, mixture of isomers) from the National Institutes for Food and Drug Control (Beijing, China); reference standards (quercetin, isorhamnetin, kaempferide, bilobalide, ginkgolides A, B, and C) (HPLC ≥ 98%) from Shanghai Yuanye Bio-Technology Co., Ltd. (Shanghai, China); HPLC-grade acetonitrile, methanol, and n-butanol from Sinopharm Chemical Reagent Co., Ltd. (Shanghai, China); Potato dextrose agar/broth (PDA/PDB) media from Qingdao Haibo Biotechnology Co., Ltd. (Qingdao, Shandong, China); and assay kits for catalase (CAT), malondialdehyde (MDA), and superoxide dismutase (SOD) activity from Solarbio Science & Technology Co., Ltd. (Product Numbers: BC0205, BC6415, BC5165) (Beijing, China).

### 2.2. Preparation of GE and Detection of GAs, Flavonoids and Ginkgolides by HPLC

GE was obtained by extracting *Ginkgo biloba* leaf powder (4 g) with 100 mL ethanol under ultrasonication (160 W, 40 kHz, 2 h), followed by filtration and rotary evaporation. The content of GAs was determined using an Agilent 1260 HPLC system (Agilent Technologies, Santa Clara, CA, USA) at 310 nm using three independent samples, each analyzed in triplicate. Separation was performed on an ODS-BP C18 column (4.6 × 250 mm, 5 μm) at 40 °C and 1.0 mL/min. The mobile phase consisted of 0.4% (*v*/*v*) phosphoric acid (A) and 100% acetonitrile (B) with the following gradient: 0–30 min, 75–90% B; 30–45 min, 90% B; 45–50 min, 90–95% B.

The degradation rate was defined as the reduction in the peak area of GAs after fermentation, and the calculation formula is as follows:

$$Degradation\;rate(\%)=\frac{A-B}{A}\times 100$$

Here, A and B represent the HPLC peak areas of GAs in the blank control group (GE only) and the fermented group, respectively.

Flavonoids (isorhamnetin, kaempferol, quercetin) and ginkgolides (bilobalide, ginkgolides A, B, C) were analyzed using an Agilent 1260 HPLC system equipped with an ODS-BP C18 column (4.6 × 250 mm, 5 μm) at 30 °C and 1.0 mL/min. The mobile phase was 0.4% (*v*/*v*) phosphoric acid (A) and methanol (B). For flavonoid analysis, detection was at 360 nm under isocratic elution with 55% B (0–45 min). For ginkgolide analysis, detection was at 222 nm under isocratic elution with 30% B (0–45 min). Degradation rates for both were calculated based on peak area comparison.

### 2.3. Isolation and Identification of GAs-Degrading Fungi

#### 2.3.1. Screening of Strains Capable of GAs Degradation

Healthy ginkgo leaves, branches, and trunks were washed with sterile deionized water. Branches and trunks were treated sequentially with 2.5% sodium hypochlorite for 60 s and 75% ethanol for 90 s; while leaves were directly disinfected in 75% ethanol for 90 s. After disinfection, tissues were cut into small pieces, inoculated onto PDA medium, and cultured at 28 °C. Hyphae growing from the tissue margins were transferred to fresh PDA for purification through multiple transfers.

Purified fungal strains were cultivated for 7 days at 28 °C under two conditions. For liquid fermentation, strains were grown in 200 mL of sterile PDB supplemented with 200 mg GE (in 2 mL methanol) with shaking at 120 rpm. For solid fermentation, strains were inoculated onto sterilized ginkgo leaf powder (4 g moistened with 8 mL sterile water) and incubated statically. Uninoculated controls were prepared for both methods.

After incubation, the fermentation products were extracted three times with 200 mL of n-butanol. Following phase separation, the supernatant was collected after removal of the culture medium and mycelial residue. The pooled organic phase was then concentrated under reduced pressure and filtered through a 0.22 μm membrane.

#### 2.3.2. Identification of the Isolated Strain

Identification of strains capable of degrading GAs via internal transcribed spacer (ITS) sequencing. Genomic DNA of the fungus was extracted using the cetyltrimethylammonium bromide (CTAB) method [34]. Mycelia were mechanically disrupted with glass beads and lysed in CTAB buffer containing β-mercaptoethanol at 65 °C. Proteins and impurities were removed by sequential extraction with phenol: chloroform: isoamyl alcohol (25:24:1) and chloroform: isoamyl alcohol (24:1). DNA was precipitated with isopropanol, treated with RNase A, washed with ethanol, and finally dissolved in nuclease-free water. The purified DNA was stored at −20 °C for subsequent use.

The ITS region was amplified by PCR using the universal primers ITS1 and ITS4 in a 25 μL reaction system containing 2× Q5 High-Fidelity Master Mix. The thermal cycling protocol comprised an initial denaturation at 98 °C for 30 s; followed by 30 cycles of denaturation at 98 °C for 10 s, annealing at 58 °C for 30 s, and extension at 72 °C for 45 s; and a final extension at 72 °C for 5 min. Successful amplification was confirmed by 1% agarose gel electrophoresis.

The target band was gel-purified and bidirectionally sequenced. PCR products were sequenced and then compared with the ITS sequences of type strains of fungal strains in the NCBI GenBank database for identification.

### 2.4. Scanning Electron Microscope Analysis

#### 2.4.1. The Influence of GE on the Microscopic Morphology of *Fusarium* sp. DLT-118

To observe GE-induced morphological adaptations in *Fusarium* sp. DLT-118 during GAs degradation, hyphal microstructure was analyzed by scanning electron microscopy (SEM). From a fresh 5-day-old PDA plate of *Fusarium* sp. DLT-118, a 1 cm^2^ plug of actively growing mycelium was aseptically excised and inoculated into 200 mL of PDB medium. The GE group received GE (1 mg/mL in methanol), while the control (CK) received an equal volume of methanol. Cultures were shaken (120 rpm) at 28 °C and harvested after 3, 5, and 7 days [35].

Fresh mycelia were fixed (room temperature, 30 min), stored at 4 °C, then rinsed with 0.1 M PBS and post-fixed with 1% osmium tetroxide (in PBS, dark, 1–2 h). After further PBS rinses, samples were dehydrated in a graded ethanol series (30%, 50%, 70%, 80%, 90%, 95%, and 100%, 15 min per step), treated with isoamyl acetate for 15 min, and subjected to critical-point drying. Dried samples were mounted on carbon tape, sputter-coated with gold for approximately 30 s, and imaged using a Hitachi SU8100 SEM (Hitachi High-Technologies, Tokyo, Japan) [36].

#### 2.4.2. Solid-State Fermentation of *Fusarium* sp. DLT-118 on the Morphology Analysis of *Ginkgo Biloba* Leaves Powder

SEM was used to compare the microstructure of *Ginkgo biloba* leaf powder before and after fermentation with *Fusarium* sp. DLT-118. This analysis linked morphological changes to GAs degradation, thereby providing direct evidence to support its potential application. Sterilized leaf powder (1 g moistened with 1 mL sterile water) was inoculated with the fungus and incubated at 28 °C for 7 days, with an uninoculated control. All samples were processed for SEM as described in Section 2.4.1 and subsequently imaged.

### 2.5. Effects of Oxidative Stress

To assess oxidative stress during GAs degradation in GE, the activities of CAT and SOD, as well as the MDA content, were measured in the mycelium of *Fusarium* sp. DLT-118. The fungus was cultured in PDB with GE (1 mg/mL) or methanol (control) at 28 °C and 120 rpm for 7 days. Mycelial biomass (0.1 g) was homogenized in 1 mL of kit-provided protein extraction buffer, followed by centrifugation (8000 rpm, 5 min). The supernatant was analyzed using commercial assay kits [37]. Data are presented as the mean of three independent experiments.

### 2.6. Non-Targeted Metabolomics Analysis of the Secondary Metabolites of Fusarium sp. DLT-118

*Fusarium* sp. DLT-118 was cultured in 200 mL of sterile PDB at 28 °C for 7 days with shaking at 120 rpm. Following incubation, the fermentation broth was extracted three times with an equal volume of n-butanol. After phase separation, the supernatant was collected upon removal of the culture medium and mycelial residue. The pooled organic phase was concentrated under reduced pressure, filtered through a 0.22 μm membrane, and subjected to subsequent metabolomic analysis.

For Liquid Chromatography-Mass Spectrometry (LC-MS) analysis, 100 μL of the sample was prepared by vortexing and centrifugation. The supernatant was analyzed using a Waters ACQUITY UPLC H-Class system (Waters Corporation, Milford, CT, USA) coupled to an AB SCIEX 5600+ TripleTOF mass spectrometer (AB SCIEX, Framingham, MA, USA). Chromatographic separation was performed on an HSS T3 column (100 mm × 2.1 mm, 1.8 μm) (Waters Corporation, Milford, CT, USA) at 40 °C with an injection volume of 4 μL. The mobile phase consisted of (A) acetonitrile and (B) 0.4% formic acid in water, delivered at 0.4 mL/min under a multi-step gradient. The mass spectrometer was operated in positive electrospray ionization (ESI+) mode with an ion spray voltage of 5500 V and a source temperature of 550 °C. Data were acquired in information-dependent acquisition (IDA) mode, collecting full-scan MS spectra (*m*/*z* 50–1200) followed by MS/MS scans of the top 15 most intense precursor ions [38].

Raw data were processed with MS-DIAL. Metabolites were identified by matching MS^1^ and MS^2^ spectra against public databases.

### 2.7. Transcriptomics Analysis

To elucidate the molecular mechanisms of fungal adaptation during GAs degradation in GE, comparative transcriptomic analysis was performed. *Fusarium* sp. DLT-118 was cultured in PDB with or without 1 mg/mL GE for 7 days (three biological replicates per group). Mycelia were harvested, flash-frozen in liquid nitrogen, and transported on dry ice. Total RNA extraction, library construction, and sequencing were conducted by Majorbio Bio-Pharm (Shanghai, China) [39]. Differentially expressed genes (DEGs) were identified with thresholds of |log_2_FC| ≥ 1 and an adjusted *p*-value (False Discovery Rate, FDR/padjust) < 0.05. Functional enrichment analyses, including Gene Ontology (GO) annotation and Kyoto Encyclopedia of Genes and Genomes (KEGG) pathway mapping, were performed on the Majorbio Cloud Platform using Goatools and SciPy, with significance set at a Bonferroni-corrected *p* < 0.05. GO functional enrichment was calculated using Goatools, while KEGG pathway enrichment analysis was implemented via the SciPy library in Python (version 1.0.0). All bioinformatic analyses were executed on the Majorbio Cloud Platform (https://cloud.majorbio.com/).

### 2.8. Metabolomics Analysis

To elucidate the metabolic changes in *Fusarium* sp. DLT-118 during the degradation of GAs in GE, metabolomic analysis was performed on samples treated identically to those used in the transcriptomic study. Metabolites were extracted from 100 mg mycelia using 800 μL of extraction solution containing L-2-chlorophenylalanine (internal standard) via cryogenic grinding and sonication [40]. After centrifugation (13,000× *g*, 4 °C, 15 min), the supernatant was analyzed. Separation was achieved on a Thermo Fisher UHPLC-Q Exactive HF-X system (Thermo Fisher Scientific, Waltham, MA, USA) with an HSS T3 column (100 mm × 2.1 mm, 1.8 μm). Mass spectrometry was performed in both positive and negative ion modes (*m/z* 50–1000) with ion spray voltages of 5000 V and 4000 V, respectively. Additional parameters were a declustering potential of 80 V, nebulizer and auxiliary gas pressures of 50 psi, a curtain gas pressure of 30 psi, an ion source temperature of 500 °C, and a collision energy range of 20–60 V. Raw data were processed using Progenesis QI (Waters Corporation, Milford, CT, USA), and metabolites were identified by matching MS/MS spectra against the HMDB (http://www.hmdb.ca/, accessed on 9 September 2025), Metlin (https://metlin.scripps.edu/, accessed on 9 September 2025), and Majorbio Cloud Platform, with six biological replicates analyzed per group.

### 2.9. Multi-Omics Integrated Analysis of Transcriptomics and Metabolomics

To identify the key functional pathways involved in GAs degradation in GE by *Fusarium* sp. DLT-118, transcriptomic and metabolomic datasets were integrated [41]. A target–pathway interaction network was constructed to visualize associations between core targets and pathways. Spearman correlation analysis was then applied to examine relationships between DEGs and differential metabolites. Significantly correlated pairs (*p* < 0.05) were selected and visualized in correlation network diagrams.

### 2.10. Antioxidant Activity Test

The antioxidant activity of GE and Fermented GE was determined using the 2,2-diphenyl-1-picrylhydrazyl (DPPH) radical scavenging assay [42]. Ascorbic acid was used as a positive control. Samples dissolved in dimethyl sulfoxide (DMSO) were mixed with 0.1 mM DPPH ethanol solution at various concentrations. After incubation in the dark at room temperature for 30 min, absorbance was measured at 517 nm.

The scavenging activity (%) was calculated as:

$$DPPH\;scavenging\;activity\;(\%)=\left(1-\frac{A_{sample}-A_{sample\;blank}}{A_{control}}\right)\times 100$$

where A_sample_ is the absorbance of the sample mixed with DPPH solution, A_sample blank_ is the absorbance of the sample without DPPH, and A_control_ is the absorbance of the DPPH solution with DMSO.

### 2.11. Cytotoxicity Evaluation of the Degradation Products of GAs

To verify the biosafety of GE after GAs degradation, the cytotoxicity of degradation products was evaluated on human normal lung epithelial (Beas-2B) and gastric mucosal (GES-1) cells using the Methylthiazolyldiphenyl-tetrazolium bromide (MTT) assay [43]. Cells were cultured in RPMI-1640 medium with 10% fetal bovine serum (FBS) and 1% penicillin/streptomycin at 37 °C under 5% CO_2_. Cells were seeded in 96-well plates at 1 × 10^4^ cells/well. After 24 h, they were treated with test samples for 48 h. Then, 20 μL of MTT solution (5 mg/mL) was added per well, followed by 4 h incubation. The formazan crystals were dissolved in 150 μL DMSO, and absorbance was measured at 490 nm. Cell viability was expressed as a percentage relative to the control.

### 2.12. Statistical Analysis

Statistical analysis of the data was performed using GraphPad Prism software (version 9.0). All analyses were carried out with three independent replicates, and results are expressed as mean ± standard deviation. Differences between groups were evaluated using one-way analysis of variance (ANOVA) followed by unpaired *t*-test for pairwise comparisons where applicable.

## 3. Results

### 3.1. Effects of Fermentation on GE and Plant Material

#### 3.1.1. Effect of Fermentation on GAs Content and Identification of the Degrading Strain

**Figure 1:**
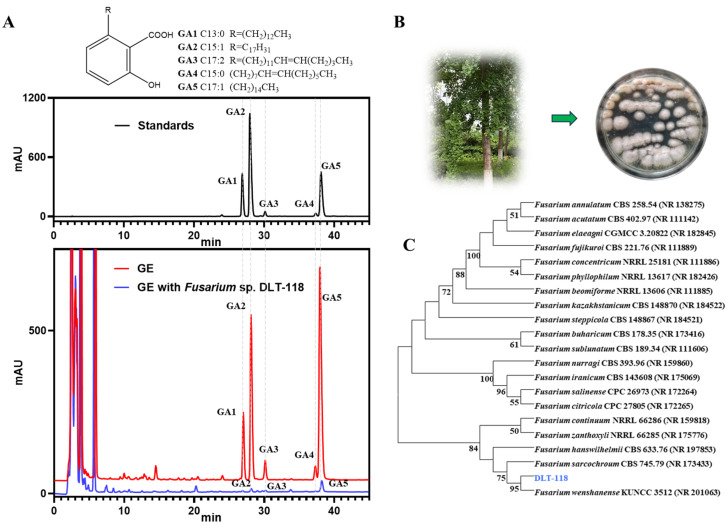
Identification of a GAs-degrading fungal strain. (**A**) The structures of GAs and HPLC analysis of GAs components at 310 nm. The black line represents the standard sample of GAs. The red and blue lines represent the unfermented GE and the GE fermented with strain DLT-118, respectively. Peaks GA1–GA5 correspond to the identified GAs monomers as GA1, C13:0; GA2, C15:1; GA3, C17:2; GA4, C15:0; GA5, C17:1. (**B**) Colony morphology of the endophytic fungus DLT-118. (**C**) Phylogenetic tree of *Fusarium* sp. DLT-118 inferred from ITS sequences using the neighbor-joining method.

From 24 endophytic fungi isolated from *Ginkgo biloba*, strain DLT-118 exhibited the highest GAs-degrading activity (Table 1). In 7-day liquid fermentation, it degraded 96.47% of total GAs (Figure 1A, Table 2). Individual GAs (C13:0, C15:1, C17:2, C15:0, and C17:1) were all significantly degraded. Solid-state fermentation also yielded a high degradation rate (95.56%), demonstrating consistent performance across both systems. During GAs degradation, major flavonoids (quercetin and kaempferol) remained stable, while ginkgolides showed varied responses. Ginkgolide B increased from 1.42 to 1.47 mg/g, ginkgolide C decreased from 5.23 to 4.04 mg/g, and bilobalide and ginkgolide A showed no obvious change.

The colony morphology on PDA was characterized by a fluffy, white mycelium producing orange-red sporodochia (Figure 1B). Based on ITS rDNA sequencing and phylogenetic analysis, DLT-118 clustered robustly with reference *Fusarium* species (Figure 1C). This strain has been deposited in the CCTCC (accession NO: M 20252976), and its ITS sequence submitted to GenBank (accession PV867279).

#### 3.1.2. Microstructure of Solid-State Fermented *Ginkgo Biloba* Leaf Powder by *Fusarium* sp. DLT-118

**Figure 2:**
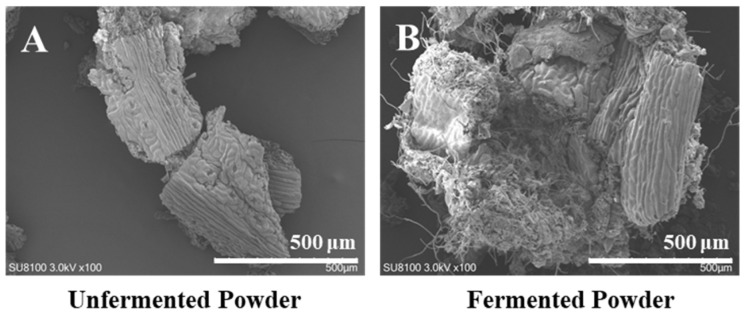
SEM of unfermented *Ginkgo biloba* leaves and *Fusarium* sp. DLT-118 solid-state fermented *Ginkgo biloba* leaves. (**A**) Unfermented *Ginkgo biloba* leaf powder. (**B**) Fermented *Ginkgo biloba* leaf powder.

SEM revealed that solid-state fermentation with *Fusarium* sp. DLT-118 significantly altered the microstructure of *Ginkgo biloba* leaf powder, transforming it from a compact, smooth form (Figure 2A) to a loose, porous structure with grooves and pores due to mycelial colonization and bio-erosion (Figure 2B). This increased surface area facilitated interfacial contact for GAs degradation, which reached 95.56% (Table 1). These results indicate that the ability of this strain to disrupt plant cell walls facilitates the release and degradation of embedded GAs.

#### 3.1.3. Analysis of Antioxidant Activity

**Figure 3:**
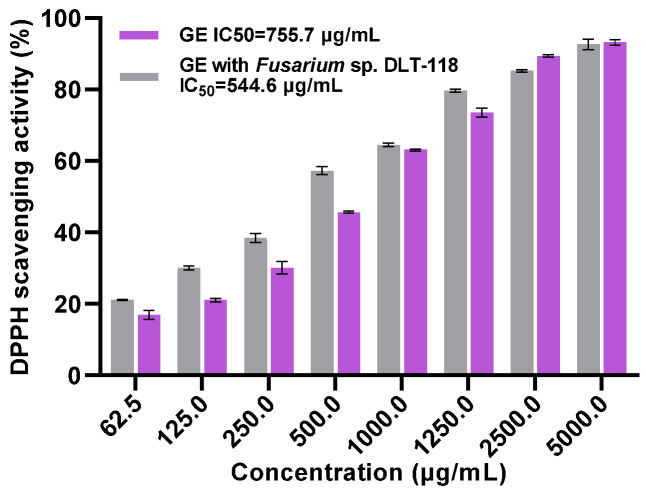
DPPH scavenging activity of GE Before and After Fermentation with *Fusarium* sp. DLT-118.

The DPPH radical scavenging assay results demonstrated a significant enhancement in the in vitro antioxidant capacity of the GE sample following the fermentation and degradation of GAs by *Fusarium* sp. DLT-118 (Figure 3). At all tested concentrations (62.5–5000.0 μg/mL), the DPPH scavenging activity of the fermented GE group was higher than that of the untreated GE group, and both groups exhibited clear dose-dependence. The calculated IC_50_ further indicated that the IC_50_ value of the original GE was 755.7 μg/mL, while after fermentation with DLT-118, the IC_50_ decreased to 544.6 μg/mL. The classic antioxidant ascorbic acid, used as a positive control, exhibited an IC_50_ of 23.04 μg/mL (Figure S14). These results confirm that fermentation with *Fusarium* sp. DLT-118 effectively improves the antioxidant activity of GE through biotransformation, providing experimental support for its potential application in functional products or antioxidant development.

#### 3.1.4. Cytotoxicity of GE Fermented by *Fusarium* sp. DLT-118

**Figure 4:**
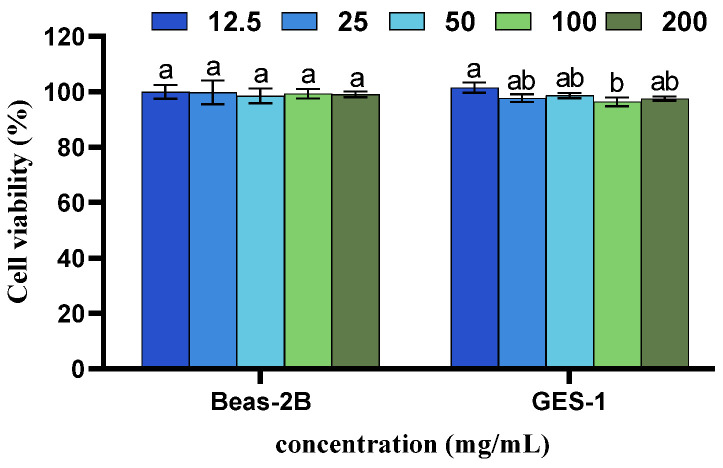
Cytotoxicity assessment of degraded GE. Different letters indicate significant difference identified by one-way ANOVA (*p* < 0.05).

To assess the cytotoxicity of GE following the degradation of its GAs by *Fusarium* sp. DLT-118, Beas-2B and GES-1 cells were used in this study. The results indicated that within the concentration range of 25–200 mg/mL, GE showed no significant toxicity toward either cell type and cell viability remained high across all tested concentrations (Figure 4). The observed low cytotoxicity is likely attributable to the efficient degradation of hazardous GAs by *Fusarium* sp. DLT-118. Therefore, this biodegradation process effectively mitigates the hazards associated with high GAs content.

### 3.2. Effects and Responses of Fusarium sp. DLT-118 During Fermentation and Degradation Processes

#### 3.2.1. Microstructural Changes in *Fusarium* sp. DLT-118 During GAs Degradation

**Figure 5:**
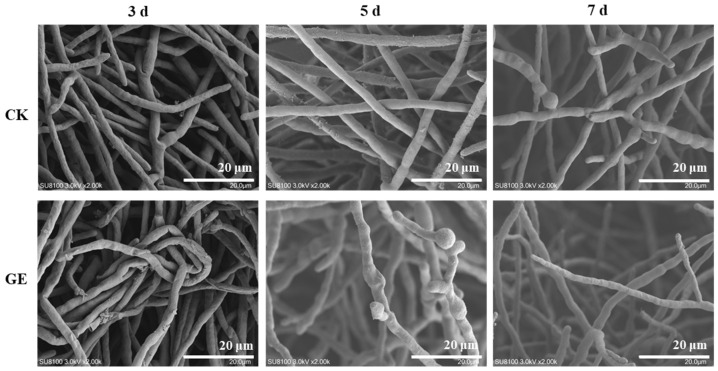
Comparison of the hyphal morphology of *Fusarium* sp. DLT-118 with and without GE treatment at 3, 5, and 7 days.

SEM analysis revealed the morphological adaptation of *Fusarium* sp. DLT-118 hyphae over time during GAs degradation in GE (Figure 5). After 3 days, GE-treated hyphae showed partial shrinkage compared to the control, indicating initial growth inhibition. By day 5, shrinkage was markedly reduced, and by day 7, it had largely disappeared. This phenotypic recovery suggests that the fungus gradually adapted to the GE environment, likely facilitated by the concurrent degradation of GAs and the consequent alleviation of toxin stress.

#### 3.2.2. Oxidative Stress Response

**Figure 6:**
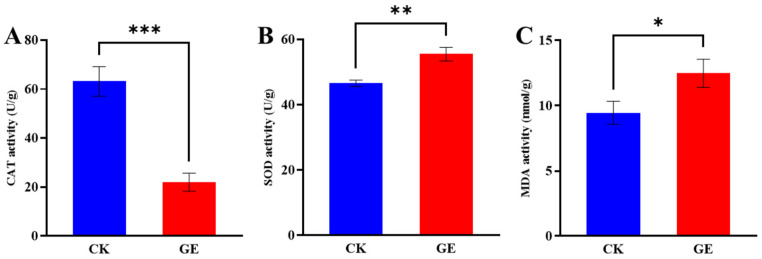
Effect of GE treatment on the oxidative stress of *Fusarium* sp. DLT-118. (**A**) CAT activity. (**B**) SOD activity. (**C**) MDA content. * *p* < 0.05, ** *p* < 0.01, *** *p* < 0.001 when compared with the CK group.

During the degradation of GAs in GE, *Fusarium* sp. DLT-118 showed significant changes in oxidative stress markers (Figure 6). Compared to the control, CAT activity decreased markedly, whereas SOD activity and MDA content increased (Figure 6A–C). The elevated MDA level indicated enhanced lipid peroxidation and oxidative damage. These findings suggest that the GAs degradation process is accompanied by oxidative stress, reflecting a dual survival strategy wherein an active detoxification response coexists with passive damage adaptation under toxin pressure.

#### 3.2.3. Chemical Diversity in the Secondary Metabolites of *Fusarium* sp. DLT-118

**Figure 7:**
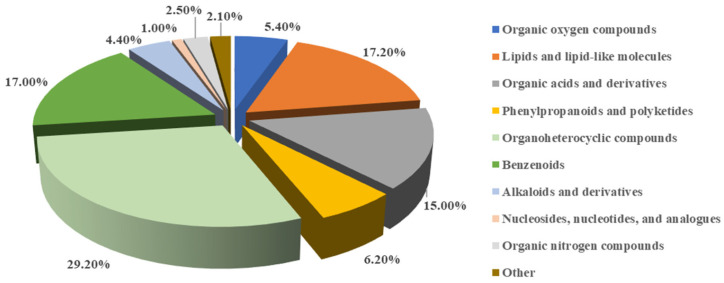
Distribution of superclass in secondary metabolites from *Fusarium* sp. DLT-118.

Non-targeted metabolomics analysis of the fermentation products of *Fusarium* sp. DLT-118 identified 681 metabolites, revealing extensive chemical diversity across multiple chemical superclass (Figure 7). Organoheterocyclic compounds constituted the largest class (199 metabolites, 29.2%), represented by compounds such as norharman and phthalic anhydride (Table S14). This was followed by lipids and lipid-like molecules (17.2%; e.g., cinobufagin, 1-monostearin), benzenoids (17.0%; e.g., N-acetyltyramine, phenylacetaldehyde), and organic acids and derivatives (15.0%; e.g., acuminatum B, betaine). Other significant classes included phenylpropanoids and polyketides (6.2%), organic oxygen compounds (5.4%), alkaloids and derivatives (4.4%), and organic nitrogen compounds (2.5%). Nucleosides, nucleotides, and analogues accounted for 1.0% of the total, with the remaining 2.1% distributed among other minor classes. This diverse metabolite profile reflects a highly active and complex secondary metabolism, underscoring the strain’s potential as a versatile biosynthetic platform.

Critically, among the 681 metabolites identified, no characteristic mycotoxins typically associated with *Fusarium* spp., including fumonisins, trichothecenes, and zearalenone, were detected among the identified compounds. This finding provides important preliminary evidence that *Fusarium* sp. DLT-118 does not produce these high-risk toxins under the experimental conditions.

#### 3.2.4. Comparative Transcriptomic Analysis

**Figure 8:**
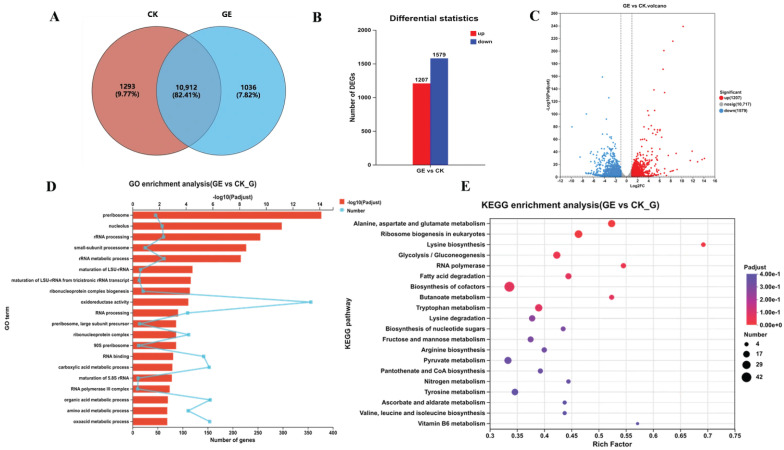
Transcriptomic analysis using mRNA of *Fusarium* sp. DLT-118 treated with GE. (**A**) Venn diagram for genes identified in control versus GE-treaded groups. (**B**) Red color shows upregulated DEGs, while blue color shows down-expressed DEGs. (**C**) Volcano plot for DEGs. Red dots represent upregulated DEGs, blue dots represent down-expressed DEGs, and gray dots represent non-DEGs. (**D**) GO enrichment of DEGs in BP and CC. (**E**) KEGG enrichment of DEGs.

Comparative transcriptomic profiling identified 12,205 and 11,948 expressed genes in *Fusarium* sp. DLT-118 under GE-treated and control conditions, respectively, during GAs degradation (Figure 8A). Applying thresholds of |log_2_Fold| ≥ 1 and FDR/padjust < 0.05, a total of 2786 differentially expressed genes (DEGs) were defined, comprising 1207 (red) downregulated and 1579 (blue) upregulated genes (Figure 8B,C).

GO enrichment analysis of DEGs highlighted key response mechanisms (Figure 8D, Table S2). In cellular component (CC), DEGs were enriched in preribosome and nucleolus terms, suggesting an impact on ribosome assembly. Related biological processes (BPs) included rRNA processing, rRNA metabolic process, maturation of LSU-rRNA, maturation of LSU-rRNA from tricistronic rRNA transcripts (SSU-rRNA, 5.8S rRNA, LSU-rRNA), ribonucleoprotein complex biogenesis, and RNA processing. Molecular function (MF) showed significant enrichment in oxidoreductase activity. Collectively, these results indicate that cells may enhance ribosome biogenesis and protein synthesis capacity to activate detoxification or repair mechanisms under GE-induced stress.

Furthermore, KEGG pathway enrichment analysis (Figure 8E and Table S3) mapped all DEGs to 123 pathways, among which five were significantly enriched, including alanine, aspartate and glutamate metabolism, ribosome biogenesis in eukaryotes, lysine biosynthesis, glycolysis/gluconeogenesis, and RNA polymerase. Together, the GO and KEGG findings support the hypothesis that *Fusarium* sp. DLT-118 upregulates ribosome biogenesis to enhance protein translation during GAs degradation in GE. Specifically, the degradation of GAs triggered the upregulation of key genes involved in nitrogen assimilation (*glnA,* which encodes glutamine synthetase; *asnB,* encoding asparagine synthetase) (Table S15), arginine and purine metabolism (*argH*, *purB*), and glutathione precursor synthesis. Ribosome biogenesis genes (*NOP56*, encoding a ribonucleoprotein involved in rRNA processing; *UTP22*) were also induced, facilitating rRNA processing and ribosome maturation to boost protein synthesis capacity. Concurrent upregulation of lysine biosynthesis genes (*LYS21,* encoding homocitrate synthase; *LYS4*, *LYS2*) and gluconeogenic enzymes (*pckA*, encoding phosphoenolpyruvate carboxykinase; *fbp)* supported detoxification and metabolic replenishment. Enrichment of the RNA polymerase pathway further indicated that transcriptional reprogramming is essential for stress adaptation (Pol I, II, III), coordinating mRNA synthesis for subsequent protein production. These adaptive mechanisms maintain the nitrogen and energy homeostasis of *Fusarium* sp. DLT-118, thereby supporting its efficient degradation of GAs in GE.

#### 3.2.5. Metabolomic Analysis

**Figure 9:**
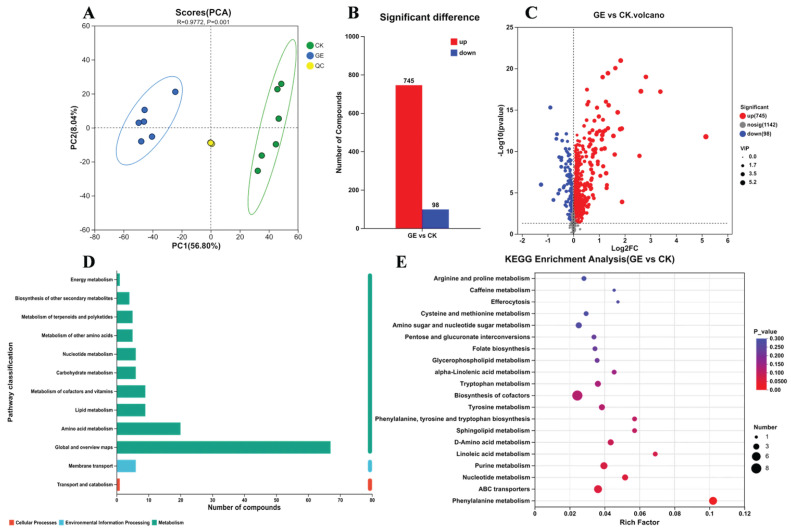
Results of metabolomics analysis. (**A**) Principal component analysis (PCA) score plot. (**B**) Bar plot of differential metabolites. (**C**) Volcano plot. (**D**) KEGG statistics plot. (**E**) KEGG enrichment bubble plot.

Principal Component Analysis (PCA) revealed distinct metabolite profiles among groups with satisfactory reproducibility (Figure 9A). Using an OPLS-DA model with thresholds of VIP > 1 and *p* < 0.05, 843 differentially expressed metabolites were screened between GE and CK groups, comprising 745 upregulated and 98 downregulated compounds (Figure 9B,C). These metabolites primarily encompassed organic acids and derivatives, lipids and lipid-like molecules, phenylpropanoids and polyketides, organoheterocyclic compounds, and organic oxygen compounds.

KEGG annotation indicated that the differentially expressed metabolites were primarily associated with global and overview maps, amino acid metabolism, lipid metabolism, cofactor and vitamin metabolism, and nucleotide metabolism (Figure 9D,E, Table S4). Enrichment analysis identified 20 significantly enriched pathways (*p* < 0.05, Figure 8E). Phenylalanine metabolism (map00360) exhibited the highest enrichment level, while purine metabolism (map00230) and the ABC transporter pathway contained the greatest number of enriched metabolites. The significant enrichment of multiple amino acid and lipid metabolism pathways further indicates that the degradation of GAs in GE triggered key biological processes, including amino acid metabolic reprogramming, nucleotide dysregulation, and lipid metabolic remodeling.

#### 3.2.6. Integrated Transcriptomic and Metabolomic Analysis

**Figure 10:**
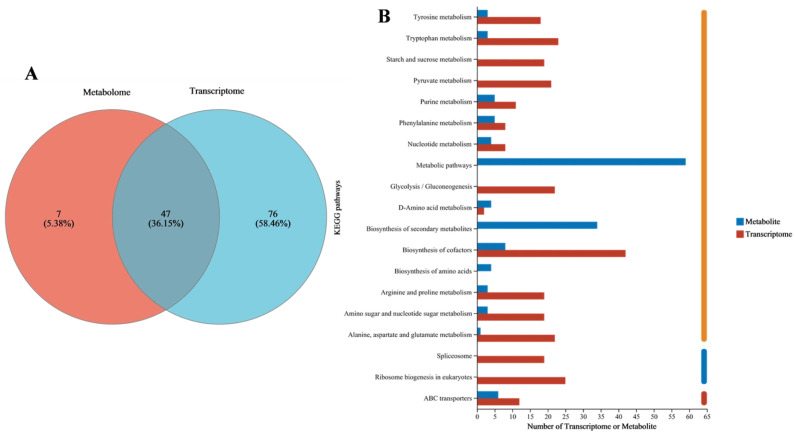
Integrated metabolomic and transcriptomic profiling. (**A**) Venn diagram of KEGG pathway annotations. (**B**) KEGG pathway analysis diagram.

The molecular mechanism underlying the degradation of GAs in GE by *Fusarium* sp. DLT-118 was revealed through integrated transcriptomic and metabolomic analyses. The co-annotation of 47 KEGG pathways (accounting for 36.15% of the intersecting pathways) indicated high functional consistency (Figure 10A). Pathway analysis revealed that the degradation of GAs in GE activated a core metabolic network (Figure 10B, Table S12), involving the remodeling of amino acid, lipid, and energy metabolism to sustain cellular activities. Coordinated changes in membrane lipid metabolism and ABC transporters suggested stress adaptation through membrane restructuring and transport adjustment. Concurrent upregulation of nucleotide metabolism and the pentose phosphate pathway reflected enhanced biosynthesis and energy demand.

## 4. Discussion

Microorganisms offer a promising approach to GAs degradation. Several bacterial and fungal strains have demonstrated degradation capabilities. Among bacteria, *Lactobacillus acidophilus*, *Lactiplantibacillus plantarum*, and *Lacticaseibacillus casei* degrade over 70% of GAs [27], while strains of *Pantoea* sp. show a degradation rate of approximately 50% [28]. For fungal species, a co-culture of *Candida tropicalis* and *Aspergillus oryzae* via solid-state fermentation of ginkgo leaf residue reduced GAs content from 14.8 to 1.5 mg/g, yielding a non-cytotoxic product [44]. *Eurotium cristatum* completely degraded GAs and improved aroma to ginkgo seed powder via liquid fermentation [29]. Furthermore, nine *Fusarium* sp. strains screened from ginkgo rhizosphere soil degraded approximately 95% of GAs, confirming the genus’s potential [28]. Notably, this study first isolated a *Fusarium* sp. strain, DLT-118, from endophytic fungi in ginkgo tree trunk, with its degradation rate in liquid culture reaching 96.47%. Through long-term symbiotic relationships with host plants, endophytic fungi have evolved efficient detoxification potential, characterized by mechanisms that are both ecologically adaptive and functionally sustainable. Taking the endophytic fungus *Serendipita indica* as an example, its inoculation can significantly enhance the remediation efficiency of *Salix suchowensis* in cadmium (Cd)-contaminated soil through multiple synergistic mechanisms, while also reshaping the structure of the rhizosphere microbial community [45]. Compared to strains isolated from open environments, endophytic fungi that have undergone long-term symbiotic coevolution with their hosts may provide more targeted, efficient, and ecologically sustainable degradation pathways based on their intrinsic metabolic adaptability to host-specific compounds. This advantage hypothesis still requires further validation through systematic comparative experiments and in-depth mechanistic studies.

*Fusarium* is an important genus of filamentous fungi, some species of which are capable of producing common mycotoxins such as zearalenone and deoxynivalenol [46]. These toxins readily contaminate staple grains like wheat and maize, as well as their processed products, not only compromising food quality and safety but also potentially causing endocrine disruption, immunosuppression, and digestive system damage in animals and humans. This poses a persistent threat to food security, trade, and public health. It is important to emphasize that *Fusarium* exhibits high functional diversity. While the genus includes well-known toxigenic and pathogenic species, it also encompasses rigorously selected, beneficial strains that are safely utilized in industrial and biotechnological applications. For example, *Fusarium venenatum* is employed in the large-scale production of mycoprotein and has been approved as a meat alternative in numerous countries [47]. *Fusarium foetens* produces the metabolite FF-C1, which alleviates metabolic dysfunction-associated steatohepatitis in mice by inhibiting intestinal ceramide synthase CerS6, thereby reducing ceramide levels [48]. Additionally, strains within this genus show considerable promise in areas such as biomanufacturing, environmental remediation, and industrial enzyme production. For the *Fusarium* sp. DLT-118 examined in this study, we conducted non-targeted metabolomics analysis and cytotoxicity tests, obtaining preliminary data in support of its safety profile. Although the fermented product showed no significant cytotoxicity, its safety still requires further evaluation through genotoxicity, immunogenicity, and allergenicity assays. It is important to note that certain species within the genus *Fusarium* are known mycotoxin producers. Therefore, the safety of this specific strain in food or feed contexts remains contingent upon rigorous future assessments.

*Fusarium* sp. DLT-118 exhibited distinct morphological adaptation strategies in solid-state and liquid fermentation systems. During solid-state fermentation, the hyphae formed a porous network within the *Ginkgo* *biloba* leaf powder, significantly increasing the contact area with the substrate and thereby promoting the release and degradation of GAs, a process likely accompanied by the decomposition of plant cell walls. In liquid fermentation, the mycelium displayed dynamic morphological changes characterized by initial contraction followed by recovery, reflecting its rapid response to environmental stress. Previous studies have indicated that microorganisms can enhance their tolerance to toxic compounds such as styrene by regulating cell morphology, maintaining membrane integrity, or increasing surface hydrophobicity [49]. These micro-scale changes observed across different fermentation systems collectively provide a crucial physiological basis for the efficient degradation of GAs by DLT-118.

*Fusarium* sp. DLT-118 employed an adaptive defense mechanism, as reflected by its oxidative stress response, to cope with stress during GAs degradation in GE, which was evidenced by a significant increase in mycelial MDA content indicating lipid peroxidation damage, induced upregulation of SOD activity, and significant inhibition of CAT activity. This response pattern between SOD and CAT activity shares similarities with the oxidative stress response of *Pleurotus ostreatus* under decabromodiphenyl ethane stress [50]. Both studies observed MDA accumulation and dynamic adjustments in the antioxidant enzyme system, demonstrating that lipid peroxidation is a common form of damage in fungi exposed to toxic substances.

Integrated omics analysis revealed that upregulation of the ribosome biogenesis gene *NOP56* enhanced ribosomal assembly and protein synthesis in *Fusarium* sp. DLT-118, thereby supporting the expression of degradation enzymes and transporters. Notably, *NOP56* is also a key proliferation factor highly expressed in various human cancers [51], mirroring its role in boosting biosynthetic capacity under stress. Enhancing ribosome biogenesis to boost translational capacity is a fundamental cellular strategy for environmental adaptation. Parallel to this, the upregulation of genes like *glnA* and *asnB* fortified the alanine, aspartate, and glutamate metabolism pathway. Similarly, in postharvest *pakchoi*, treatment with lactopeptide and ectoine upregulated *asnB*, increased L-aspartate and L-glutamate accumulation, and delayed yellowing [52]. This indicates that enhancing aspartate-family amino acid metabolism is a conserved strategy across organisms to meet nitrogen demands and sustain biosynthesis under stress. During GAs degradation, the gene *pckA* in the gluconeogenesis pathway was upregulated in *Fusarium* sp. DLT-118. This aligns with findings in a fungal–bacterial symbiotic system, where *pckA* upregulation in bacteria supported proliferation under nutrient stress [53]. As a key enzyme in the gluconeogenesis/glycolysis pathway, the upregulation of *pckA* plays a crucial role in different stress responses.

Additionally, this study observed coordinated changes in the ABC transporter pathway and membrane lipid metabolism, indicating that *Fusarium* sp. DLT-118 remodels its transport and membrane systems during the degradation of GAs in GE. This aligns with the induction of ABC transporters in Kentucky bluegrass under cadmium stress, a conserved detoxification response across kingdoms [54]. Thus, ABC transporters likely play a pivotal role in the fungal degradation of GAs by enhancing toxin efflux. Notably, lipid metabolism was one of the most significantly enriched pathways in both the transcriptomic and metabolomic datasets. Integrated omics analysis, particularly the accumulation of specific lipid-like metabolites, suggests that the degradation of GAs may involve extensive membrane remodeling or the utilization of lipid-like intermediates. From a chemical structural perspective, the long aliphatic side chains of GAs are highly hydrophobic, and the intermediates generated during their degradation may be difficult to retain or transport stably within the cell. These side chains may be partially metabolized via oxidation pathways and subsequently integrated into the cellular lipid metabolic network. This lipid-centric adaptive response, combined with the induction of ABC transporters, which are likely responsible for the active efflux of hydrophobic intermediates, may together constitute a coordinated cellular strategy to cope with lipophilic stress and enhance degradation efficiency. This mechanistic hypothesis remains to be experimentally validated.

Several significantly upregulated metabolites were identified as potential direct derivatives of GAs (Table S16). The detection of 2-hydroxy-4-pentadecylbenzoic acid (CSA ID: 74009-48-6), which shares the core structure of GA2 (C15:1) but possesses a saturated alkyl side chain, points to reductive modification of the parent compound. Furthermore, the accumulation of 5-(12-heptadecenyl)-1,3-benzenediol (CSA ID: 103462-06-2), featuring a modified alkyl side chain and an additional phenolic hydroxyl group, suggests the occurrence of side-chain oxidation and aromatic ring hydroxylation. Together, the presence of these compounds provides molecular-level evidence supporting the active biodegradation of the GAs scaffold. The metabolites identified based on non-targeted metabolomics can be further confirmed by subsequent LC-MS/MS analysis targeting the degradation intermediates of GAs standard compounds [55]. By elucidating the mass spectrometry fragmentation patterns of these compounds, it will help verify their chemical structures and systematically clarify the associated biodegradation pathways.

Although fermentation with *Fusarium* sp. DLT-118 had little effect on flavonoids, it significantly enhanced the overall antioxidant capacity of GE. Existing studies suggest that microbial fermentation can improve bioactivity by increasing polyphenol content and generating new antioxidant compounds [56]. Although the expression of the phenylalanine metabolic pathway is upregulated during fermentation, its functional link with polyphenol synthesis requires further elucidation through subsequent targeted experiments. Future work could focus on the quantitative analysis of polyphenols and antioxidant metabolites in samples before and after fermentation, as well as the structural identification of newly formed antioxidant components.

To advance the endophytic fungus *Fusarium* sp. DLT-118 from efficient laboratory degradation to industrial application, future research should concentrate on three areas. First, in-depth mechanistic studies are required. These should include refining strain classification through multi-locus sequencing of housekeeping genes to improve phylogenetic resolution, functionally validating key candidate genes (e.g., *glnA*, *asnB*, *pckA*, *NOP56*), and integrating metabolomic techniques such as LC-MS/MS to systematically profile degradation intermediates. Together, these efforts will help elucidate the complete biodegradation pathway and intermediate metabolism of GAs. Second, a comprehensive toxicological evaluation framework must be established to systematically assess the potential safety risks posed by the degradation products and the secondary metabolites of *Fusarium* sp. DLT-118. Third, the production process should be optimized by exploring diverse fermentation strategies and co-culture approaches. Progress in these areas will lay a solid foundation for the industrial application of this strain.

## 5. Conclusions

This study isolated the endophytic fungus *Fusarium* sp. DLT-118 from *Ginkgo biloba*. It degraded 96.47% of GAs in GE and enhanced its antioxidant activity, reducing the DPPH IC_50_ from 755.7 to 544.6 μg/mL. SEM revealed key morphological adaptations during GAs degradation. The strain also mounted an oxidative stress response to counteract reactive oxygen species induced by GE. Integrated multi-omics analysis demonstrated that GE stress triggered a comprehensive metabolic remodeling in *Fusarium* sp. DLT-118, reprogramming amino acid, lipid, and energy metabolism to fuel the degradation process, while adapting membrane systems and transport functions. Critically, the resulting degradation products exhibited no significant cytotoxicity toward Beas-2B or GES-1 cells. While the complete degradation pathway and transformation products require further clarification, this work establishes microbial fermentation as a promising strategy for GAs detoxification and provides a valuable microbial resource for developing safer Ginkgo-based products. These findings indicate that this endophytic fungus shows potential as a candidate for the degradation of GAs. With further development, it may support the production of safer and functionally enhanced ingredients, which could be suitable for nutraceutical and functional food applications.

## Figures and Tables

**Table 1:** Degradation of GAs by endophytic fungi from *Ginkgo biloba*.

The Serial Numbers of Fungi	Source	Strain Photograph	GAs Degradation Rate		The Serial Numbers of Fungi	Source	Strain Photograph	GAs Degradation Rate	
			Liquid Fermentation	Solid Fermentation				Liquid Fermentation	Solid Fermentation
DLT-116	Ginkgo tree trunk	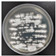	81.38%	<10.00%	DLT-128	Ginkgo tree trunk	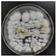	<10%	<10%
DLT-117	Ginkgo leaf	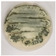	41.09%	<10.00%	DLT-130	Ginkgo tree trunk	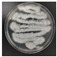	35.96%	67.97%
DLT-118	Ginkgo tree trunk	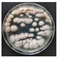	96.47%	95.56%	DLT-131	Ginkgo tree trunk	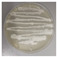	<10.00%	<10.00%
DLT-119	Ginkgo tree trunk	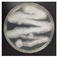	<10.00%	<10.00%	DLT-132	Ginkgo leaf	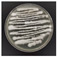	<10.00%	16.10%
DLT-120	Ginkgo tree trunk	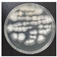	56.53%	80.65%	DLT-133	Ginkgo tree branches	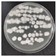	10.37%	<10.00%
DLT-121	Ginkgo tree branches	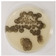	<10.00%	<10.00%	DLT-134	Ginkgo tree branches	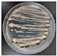	<10.00%	<10.00%
DLT-122	Ginkgo tree trunk	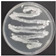	<10.00%	<10.00%	DLT-136	Ginkgo tree branches	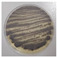	<10.00%	<10.00%
DLT-123	Ginkgo tree trunk	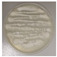	43.47%	<10.00%	DLT-137	Ginkgo tree trunk	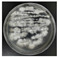	34.43%	<10.00%
DLT-124	Ginkgo tree trunk	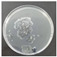	<10.00%	<10.00%	DLT-138	Ginkgo tree trunk	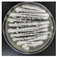	43.0%	<10.00%
DLT-125	Ginkgo tree trunk	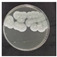	35.12%	<10.00%	DLT-139	Ginkgo tree trunk	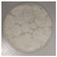	<10.00%	<10.00%
DLT-126	Ginkgo tree trunk	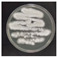	71.42%	<10.00%	DLT-140	Ginkgo leaf	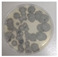	<10.00%	33.35%
DLT-127	Ginkgo tree trunk	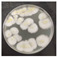	56.78%	25.57%	DLT-141	Ginkgo tree trunk	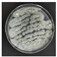	<10.00%	<10.00%

**Table 2:** Main components of GE before and after treatment with *Fusarium* sp. DLT-118.

Category	Components	GE (mg/g)	GE with *Fusarium* sp. DLT-118 (mg/g)
Ginkgolic acids	C13:0	1.43 ± 0.02 a	0.14 ± 0.02 b
	C15:1	3.69 ± 0.07 a	0.09 ± 0.01 b
	C17:2	0.61 ± 0.08 a	0.09 ± 0.01 b
	C15:0	0.35 ± 0.05 a	0.02 ± 0.01 b
	C17:1	8.21 ± 0.98 a	0.40 ± 0.07 b
Flavonoids	isorhamnetin	<0.01	<0.01
	kaempferol	0.58 ± 0.08 a	0.51 ± 0.07 a
	quercetin	0.19 ± 0.05 a	0.19 ± 0.03 a
Ginkgolides	bilobalide	39.40 ± 3.35 a	35.90 ± 2.12 a
	ginkgolide A	9.02 ± 0.58 a	8.35 ± 0.91 a
	ginkgolide B	1.42 ± 0.02 a	1.47 ± 0.02 b
	ginkgolide C	5.23 ± 0.12 a	4.04 ± 0.13 b

The letters “a” and “b” indicate the results of significance analysis. Values sharing the same letter within a column are not significantly different, while those labeled with different letters are statistically different.

## Data Availability

The original contributions presented in this study are included in the article/Supplementary Material. Further inquiries can be directed to the corresponding authors.

## Supplementary Materials

The following supporting information can be downloaded at: https://www.mdpi.com/article/10.3390/foods15071247/s1. The Supplementary material for this article can be found online at: Figures S1 and S2: HPLC chromatograms of GE before and after fungal treatment. Figure S3: Photographs of the fungal strain *Fusarium* sp. DLT-118. Table S1: BLAST alignment results for *Fusarium* sp. DLT-118. Tables S2 and S3 list DEGs from GO and KEGG enrichment analyses in transcriptomics. Tables S4–S10 provide comprehensive metabolomic data, including KEGG annotations and enrichment statistics for global metabolism and specific pathways. Tables S11–S13: summarize the integrated transcriptomic and metabolomic analysis, including pathway annotation and Venn diagram details. Figures S4–S8: KEGG pathway enrichment maps based on transcriptomic data. Figures S9 and S10: Effects of *Fusarium* sp. DLT-118 on flavonoids and ginkgolides content in GE. Figures S11–S13: Standard curves for quantifying GAs, flavonoids, and ginkgolides. Figure S14: DPPH scavenging activity of Ascorbic acid. Table S14: Identification results of metabolites from the fermentation products of *Fusarium* sp. DLT-118 by non-targeted metabolomics. Table S15: Annotation details of differentially expressed unigenes. Table S16: Complete list of differentially expressed metabolites identified in the metabolomic analysis.

## Abbreviations

The following abbreviations are used in this manuscript:

GAs	Ginkgolic acids
GE	*Ginkgo biloba* leaf extract
PDA	Potato dextrose agar
PDB	Potato dextrose broth
CAT	Catalase
MDA	Malondialdehyde
SOD	Superoxide dismutase
SEM	Scanning electron microscope
DEGs	Differentially expressed genes
FDR	False discovery rate
GO	Gene Ontology
KEGG	Kyoto Encyclopedia of Genes and Genomes
MTT	Methylthiazolyldiphenyl-tetrazolium bromide
FBS	Fetal bovine serum
DMSO	Dimethyl sulfoxide
CC	Cellular component
BP	Biological process
MF	Molecular function
PCA	Principal Component Analysis
HPLC	High-Performance Liquid Chromatography
LC-MS	Liquid Chromatography-Mass Spectrometry
MS/MS	Tandem Mass Spectrometry
ESI	Electrospray Ionization
