# Supplementary material for: Ginkgolic Acids Degradation by the Ginkgo biloba Endophytic Fungus Fusarium sp. DLT-118
Source: Foods. 2026 Apr 6;15(7):1247. doi: 10.3390/foods15071247 (PMC13073844; doi:10.3390/foods15071247)
Supplement: Supplementary file 1 [file foods-15-01247-s001.zip › foods-4203978-supplementary.pdf]

Supplementary Material

Ginkgolic Acids Degradation by the *Ginkgo biloba* Endophytic Fungus *Fusarium* sp. DLT-118

Luting Dai [1, 2,3], Zhifang Yu[3], Youxing Zhao [2,*], Yi Zheng [1,*]

[1] School of Food Science and Health, Jiangsu Agri-animal Husbandry Vocational College, Taizhou, 225300, China

[2] Key Laboratory of Research and Development of Natural Product from Li Folk Medicine of Hainan Province & National Key Laboratory for Tropical Crop Breeding, Institute of Tropical Bioscience and Biotechnology, Chinese Academy of Tropical Agricultural Sciences, Haikou 571101, China

[3] College of Food Science and Technology, Nanjing Agricultural University, Nanjing 210095, China

*** Correspondence:**

Youxing Zhao zhaoyouxing@catasitbb.cn (Y.Z.);

Yi Zheng 1997010030@jsahvc.edu.cn (Y.Z.)

Table of Contents

Fig. S1. HPLC chromatogram of GE (at 310 nm). 3
Fig. S2. HPLC chromatogram of GE treated with *Fusarium* sp. DLT-118 (at 310 nm). 4
Fig. S3. Photographs of the *Fusarium* sp. DLT-118 fungal strain........5
Table S1. BLAST alignment results of *Fusarium* sp. DLT-118 against the NCBI database. 6
Table S2. DEGs in GO enrichment analysis........7
Table S3. DEGs in KEGG enrichment analysis........10
Table S4. KEGG annotation statistics (Metabolism) 13
Table S5. Metabolites List (Metabolites)-Global and overview maps........15
Table S6. Metabolites List (Metabolites)-amino acid metabolism........18
Table S7. Metabolites List (Metabolites)-lipid metabolism........21
Table S8. Metabolites List (Metabolites)-cofactor and vitamin metabolism 23
Table S9. Metabolites List (Metabolites)-nucleotide metabolism........25
Table S10. KEGG enrichment statistics (Metabolism) 26
Table S11. KEGG annotation statistics from the integrated analysis........28
Table S12. Venn diagram details table........31
Table S13. KEGG pathway annotation statistics table 37
Fig. S4. KEGG enrichment of alanine, aspartate, and glutamate pathway based on transcriptomic data in *Fusarium* sp. DLT-118........40
Fig. S5. KEGG enrichment of ribosome biogenesis in eukaryotes pathway based on transcriptomic data in *Fusarium* sp. DLT-118........41
Fig. S6. KEGG enrichment of lysine biosynthesis pathway based on transcriptomic data in *Fusarium* sp. DLT-118 42
Fig. S7. KEGG enrichment of glycolysis/gluconeogenesis pathway based on transcriptomic data in *Fusarium* sp. DLT-118 43
Fig. S8. KEGG enrichment of RNA polymerase pathway based on transcriptomic data in *Fusarium* sp. DLT-118........44
Fig. S9. Effect of *Fusarium* sp. DLT-118 on the flavonoids content in GE (360 nm) 45
Fig. S10. Effect of *Fusarium* sp. DLT-118 on the ginkgolides content in GE (222 nm)........46
Fig. S11. Standard curves for the quantification of ginkgolic acids 47
Fig. S12. Standard curves for the quantification of flavonoids........47
Fig. S13. Standard curves for the quantification of ginkgolides........48
Fig. S14. DPPH scavenging activity of Ascorbic acid........48
Table S14 Identification results of metabolites from the fermentation products of *Fusarium* sp. DLT-118 by non-targeted metabolomics 49
Table S15 Annotation details of differentially expressed unigenes........56
Table S16 List of differentially expressed metabolites identified in the metabolomic analysis.....60

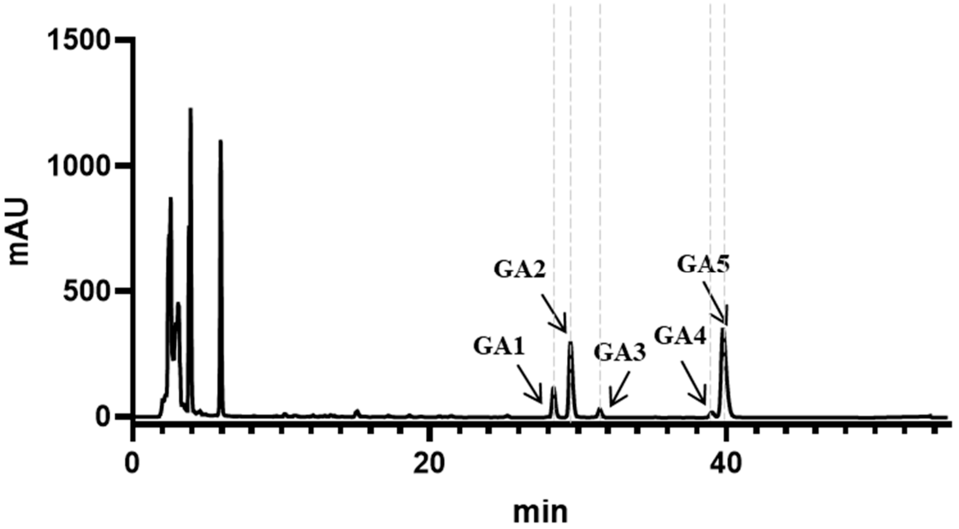

Fig. S1. HPLC chromatogram of GE (at 310 nm).

Peaks GA1–GA5 correspond to the identified GA monomers as GA1, C13:0; GA2, C15:1; GA3, C17:2; GA4, C15:0; GA5, C17:1.

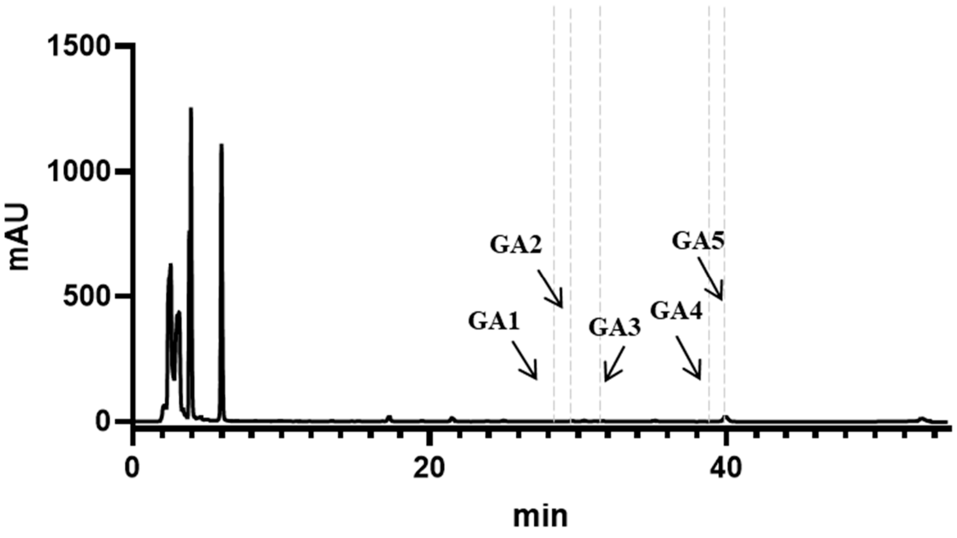

Fig. S2. HPLC chromatogram of GE treated with *Fusarium* sp. DLT-118 (at 310 nm).

Peaks GA1–GA5 correspond to the identified GA monomers as GA1, C13:0; GA2, C15:1; GA3, C17:2; GA4, C15:0; GA5, C17:1.

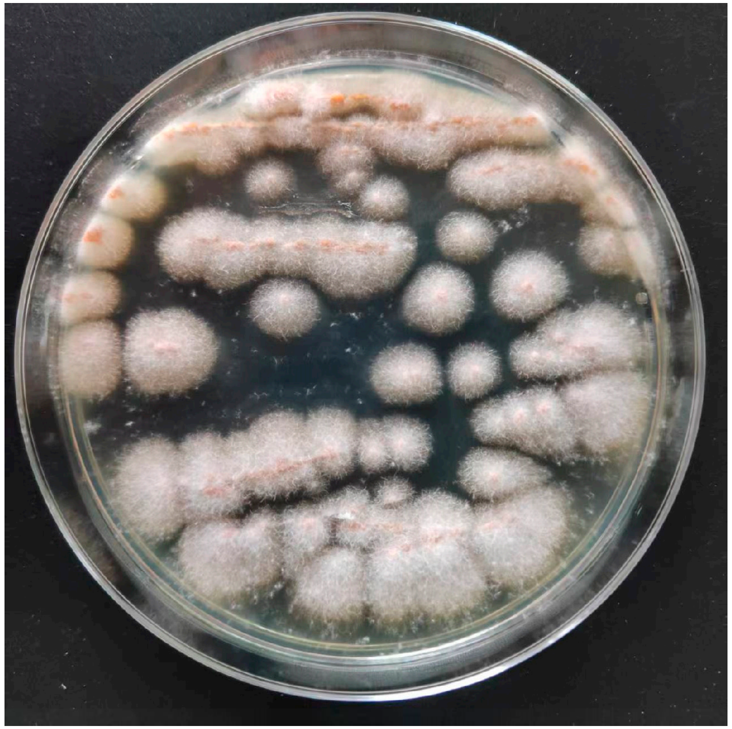

Fig. S3. Photographs of the *Fusarium* sp. DLT-118 fungal strain

Table S1. BLAST alignment results of *Fusarium* sp. DLT-118 against the NCBI database.

ITS gene sequence	DNA identification results	Percent Identity
GGGGGATCTACTGATCCGAGGTCACATTCAGAAGTTGGGGGTTTAAC<br>GGCTTGGCCGCGCCGCGTACCAGTTGCGAGGTGTTATCTACTACGCAA<br>TGGAAGCTGCAGCGTGACCGCCACTAGATTTCGGGGCCGGGCTGACT<br>GGCAGCCCGATCCCCAACACCAAACCCGGGGGTTTGAGGGTTGAAAT<br>GACGCTCGAACAGGCATGCCCGCCAGAATACTGGCGGGCGCAATGTG<br>CGTTCAAAGATTCGATGATTCACTGAATTCTGCAATTCACATTACTTA<br>TCGCATTTTGCTGCGTTCTTCATCGATGCCAGAACCAAGAGATCCGTT<br>GTTGAAAGTTTTGATTTATTTGTTTGTGTTACTCAGAAGATACACTAA<br>ATACAATAGAGTTTGGGTCCTCTGGCGGGCCGTCCCGTTTTACGGGGC<br>GCGGGCTGATCCGCCGAGGCAACAATAAGGTATGTTCACAGGGGTTT<br>GGGAGTTGTAAACTCGGTAATGATCCCTCCGCAGGCCCCCCCTACGG<br>AATC	*Fusarium* sp.	99.23%

Table S2. DEGs in GO enrichment analysis

Number	GO ID	Term Type	Description	Ratio_in_study	Ratio_in_pop	Rich factor	Pvalue	Padjust
46	GO:0030684	CC	preribosome	46/2030	60/8479	0.766667	8.30E-18	7.31E-15
59	GO:0005730	CC	nucleolus	59/2030	98/8479	0.602041	1.51E-14	6.63E-12
62	GO:0006364	BP	rRNA processing	62/2030	108/8479	0.574074	7.10E-14	2.69E-10
25	GO:0032040	CC	small-subunit processome	25/2030	30/8479	0.833333	1.05E-11	3.09E-09
63	GO:0016072	BP	rRNA metabolic process	63/2030	118/8479	0.533898	4.12E-12	7.80E-09
16	GO:0000470	BP	maturation of LSU-rRNA	16/2030	19/8479	0.842105	5.05E-08	3.27E-05
13	GO:0000463	BP	maturation of LSU-rRNA from tricistronic rRNA transcript (SSU-rRNA, 5.8S rRNA, LSU-rRNA)	13/2030	14/8479	0.928571	8.99E-08	4.37E-05
21	GO:0022613	BP	ribonucleoprotein complex biogenesis	21/2030	30/8479	0.7	1.20E-07	5.07E-05
356	GO:0016491	MF	oxidoreductase activity	356/2030	1165/8479	0.305579	2.74E-08	6.64E-05
110	GO:0006396	BP	RNA processing	110/2030	305/8479	0.360656	1.44E-06	0.000391
14	GO:0030687	CC	preribosome, large subunit precursor	14/2030	19/8479	0.736842	6.52E-06	0.000549
112	GO:1990904	CC	ribonucleoprotein complex	112/2030	321/8479	0.34891	7.00E-06	0.000549
12	GO:0030686	CC	90S preribosome	12/2030	15/8479	0.8	7.48E-	0.000549

							06	
142	GO:0003723	MF	RNA binding	142/2030	411/8479	0.345499	7.52E-07	0.000911
153	GO:0019752	BP	carboxylic acid metabolic process	153/2030	464/8479	0.329741	5.47E-06	0.001035
11	GO:0000460	BP	maturation of 5.8S rRNA	11/2030	13/8479	0.846154	6.92E-06	0.001139
10	GO:0005666	CC	RNA polymerase III complex	10/2030	12/8479	0.833333	2.46E-05	0.001668
155	GO:0006082	BP	organic acid metabolic process	155/2030	479/8479	0.323591	1.62E-05	0.00227
111	GO:0006520	BP	amino acid metabolic process	111/2030	323/8479	0.343653	1.88E-05	0.002524
154	GO:0043436	BP	oxoacid metabolic process	154/2030	476/8479	0.323529	1.93E-05	0.002524

Note:
Number: Number of unigenes/transcripts enriched in this GO term.
GO ID: Corresponding ID in the Gene Ontology database.
Term Type: The three main branches of GO: BP (Biological Process), CC (Cellular Component), and MF (Molecular Function).
Description: Functional description of the GO term.
Ratio in study: Proportion of the GO term in the target gene set. The numerator is the number of genes/transcripts in the target set enriched for that GO term, and the denominator is the total number of annotated genes/transcripts in the target set.
Ratio in pop: Proportion of the GO term in the background set of all genes/transcripts. The numerator is the number of all genes/transcripts in the background set enriched for that GO term, and the denominator is the total number of annotated genes/transcripts in the background set.
Rich factor: Ratio of the number of genes/transcripts enriched for the GO term in the target set to the number of annotated genes/transcripts in the background set. A higher value indicates a greater degree of enrichment.

Pvalue: Uncorrected p-value representing the statistical significance of the enrichment result. A smaller p-value indicates stronger statistical significance.

Table S3. DEGs in KEGG enrichment analysis

Num	Pathway id	Description	Ratio_in_study	Ratio_in_pop	Rich factor	Pvalue	Padjust	First Category	Second Category
22	map00250	Alanine, aspartate and glutamate metabolism	22/920	42/3940	0.52381	3.87E-05	0.004762	Metabolism	Amino acid metabolism
25	map03008	Ribosome biogenesis in eukaryotes	25/920	54/3940	0.462963	0.000162	0.009989	Genetic Information Processing	Translation
9	map00300	Lysine biosynthesis	9/920	13/3940	0.692308	0.000564	0.023121	Metabolism	Amino acid metabolism
22	map00010	Glycolysis / Gluconeogenesis	22/920	52/3940	0.423077	0.001773	0.043608	Metabolism	Carbohydrate metabolism
12	map03020	RNA polymerase	12/920	22/3940	0.545455	0.001486	0.045689	Genetic Information Processing	Transcription
16	map00071	Fatty acid degradation	16/920	36/3940	0.444444	0.004081	0.071712	Metabolism	Lipid metabolism
42	map01240	Biosynthesis of cofactors	42/920	125/3940	0.336	0.005262	0.071913	Metabolism	Global and overview maps
11	map00650	Butanoate metabolism	11/920	21/3940	0.52381	0.003574	0.073268	Metabolism	Carbohydrate metabolism
23	map00380	Tryptophan metabolism	23/920	59/3940	0.389831	0.004949	0.076088	Metabolism	Amino acid metabolism
17	map00310	Lysine degradation	17/920	45/3940	0.377778	0.020564	0.252937	Metabolism	Amino acid metabolism
10	map01250	Biosynthesis of nucleotide sugars	10/920	23/3940	0.434783	0.025797	0.288461	Metabolism	Global and overview maps
15	map00051	Fructose and	15/920	40/3940	0.375	0.030721	0.290664	Metabolism	Carbohydrate

		mannose metabolism							metabolism
12	map00220	Arginine biosynthesis	12/920	30/3940	0.4	0.030705	0.314724	Metabolism	Amino acid metabolism
21	map00620	Pyruvate metabolism	21/920	63/3940	0.333333	0.044943	0.325177	Metabolism	Carbohydrate metabolism
11	map00770	Pantothenate and CoA biosynthesis	11/920	28/3940	0.392857	0.043217	0.332229	Metabolism	Metabolism of cofactors and vitamins
8	map00910	Nitrogen metabolism	8/920	18/3940	0.444444	0.03906	0.34317	Metabolism	Energy metabolism
18	map00350	Tyrosine metabolism	18/920	52/3940	0.346154	0.042702	0.350158	Metabolism	Amino acid metabolism
7	map00053	Ascorbate and aldarate metabolism	7/920	16/3940	0.4375	0.057319	0.371062	Metabolism	Carbohydrate metabolism
7	map00290	Valine, leucine and isoleucine biosynthesis	7/920	16/3940	0.4375	0.057319	0.371062	Metabolism	Amino acid metabolism
4	map00750	Vitamin B6 metabolism	4/920	7/3940	0.571429	0.056165	0.383795	Metabolism	Metabolism of cofactors and vitamins

Note:
Number: Number of genes/transcripts enriched in this pathway;
Pathway ID: Pathway accession identifier;
Description: Name of the pathway;
Ratio_in_study: Proportion of this KEGG pathway in the target gene set. The numerator is the number of unigenes/transcripts enriched in this pathway within the target set, and the denominator is the total number of unigenes/transcripts in the target set annotated with KEGG terms;

Ratio_in_pop: Proportion of this KEGG pathway in the background set of all unigenes/transcripts. The numerator is the number of unigenes/transcripts enriched in this pathway within the background set, and the denominator is the total number of background unigenes/transcripts annotated with KEGG terms;

Rich factor: Ratio of the number of genes/transcripts enriched in the pathway (Sample number) to the number of annotated genes/transcripts (Background number). A higher Rich factor indicates a greater degree of enrichment.

Table S4. KEGG annotation statistics (Metabolism)

First Category	Second Category	Pathway ID	Description	GE_vs_CK_G numbers	GE_vs_CK_G_up numbers	GE_vs_CK_G_down numbers
Metabolism	Amino acid metabolism	map00310	Lysine degradation	17	10	7
Metabolism	Metabolism of other amino acids	map00470	D-Amino acid metabolism	2	1	1
Metabolism	Lipid metabolism	map00071	Fatty acid degradation	16	10	6
Metabolism	Global and overview maps	map01240	Biosynthesis of cofactors	42	21	21
Metabolism	Amino acid metabolism	map00250	Alanine, aspartate and glutamate metabolism	22	15	7
Metabolism	Lipid metabolism	map00061	Fatty acid biosynthesis	3	1	2
Metabolism	Metabolism of cofactors and vitamins	map00790	Folate biosynthesis	2	1	1
Metabolism	Metabolism of other amino acids	map00410	beta-Alanine metabolism	9	6	3
Metabolism	Carbohydrate metabolism	map00650	Butanoate metabolism	11	9	2
Metabolism	Metabolism of cofactors and vitamins	map00860	Porphyrin metabolism	4	4	0
Metabolism	Energy metabolism	map00710	Carbon fixation by Calvin cycle	7	3	4

Metabolism	Amino acid metabolism	map00300	Lysine biosynthesis	9	7	2
Metabolism	Metabolism of cofactors and vitamins	map00780	Biotin metabolism	4	1	3
Metabolism	Metabolism of cofactors and vitamins	map00785	Lipoic acid metabolism	3	3	0
Metabolism	Amino acid metabolism	map00260	Glycine, serine and threonine metabolism	16	9	7
Metabolism	Biosynthesis of other secondary metabolites	map00261	Monobactam biosynthesis	1	0	1
Metabolism	Carbohydrate metabolism	map00660	C5-Branched dibasic acid metabolism	2	2	0
Metabolism	Amino acid metabolism	map00380	Tryptophan metabolism	23	11	12
Metabolism	Metabolism of other amino acids	map00460	Cyanoamino acid metabolism	11	4	7

Table S5. Metabolites List (Metabolites)-Global and overview maps

ID	Metabolite	KEGG Pathway Description	Formula	HMDB Class
pos_227	4-Hydroxyproline	Metabolic pathways; D-Amino acid metabolism; Arginine and proline metabolism; ABC transporters	C5H9NO3	Carboxylic acids and derivatives
pos_237	D-Ala-D-Ala	Metabolic pathways; D-Amino acid metabolism	C6H12N2O3	Carboxylic acids and derivatives
pos_282	Guanosine 3'-Monophosphate	Metabolic pathways; Purine metabolism	C10H14N5O8P	Ribonucleoside 3"-phosphates
pos_326	Pyridoxal	Metabolic pathways; Vitamin B6 metabolism; Biosynthesis of cofactors	C8H9NO3	Pyridines and derivatives
pos_470	D-Altro-D-Manno-Heptose	Metabolic pathways; Biosynthesis of nucleotide sugars	C7H14O7	Organooxygen compounds
pos_566	L-Dopa	Metabolic pathways; Biosynthesis of secondary metabolites; Tyrosine metabolism	C9H11NO4	Carboxylic acids and derivatives

pos_578	Sedoheptulose	Metabolic pathways; Pentose phosphate pathway	C7H14O7	Organooxygen compounds
pos_606	N2-Succinyl-L-Ornithine	Metabolic pathways; Arginine and proline metabolism	C9H16N2O5	Carboxylic acids and derivatives
pos_1968	Ginkgolide A	Diterpenoid biosynthesis; Biosynthesis of secondary metabolites	C20H24O9	Prenol lipids
pos_2224	Beta-Farnesene	Biosynthesis of secondary metabolites; Sesquiterpenoid and triterpenoid biosynthesis	C15H24	Prenol lipids
pos_2363	Stearidonic Acid	alpha-Linolenic acid metabolism; Biosynthesis of secondary metabolites	C18H28O2	Fatty Acyls
pos_2608	Chlorophyllide A	Metabolic pathways; Biosynthesis of secondary metabolites; Porphyrin metabolism	C35H34N4O5-2	Tetrapyrroles and derivatives
pos_2644	Neomycin	Biosynthesis of secondary metabolites	C23H46N6O13	Organooxygen compounds
pos_4776	Kaempferol	Metabolic pathways; Biosynthesis of secondary metabolites	C15H10O6	Flavonoids

pos_4781	Chitobiose	Metabolic pathways; Amino sugar and nucleotide sugar metabolism; ABC transporters	$C_{15}H_{26}N_2O_{12}$	Organooxygen compounds
pos_4812	Quercetin	Metabolic pathways; Biosynthesis of secondary metabolites	$C_{15}H_{10}O_7$	Flavonoids
pos_5270	Tyramine	Metabolic pathways; Methane metabolism; Biosynthesis of secondary metabolites; Tyrosine metabolism; Biosynthesis of cofactors	$C_8H_{11}NO$	Benzene and substituted derivatives
pos_5377	Abscisic Acid	Metabolic pathways; Carotenoid biosynthesis; Biosynthesis of secondary metabolites	$C_{15}H_{20}O_4$	Prenol lipids
pos_5922	Dehydroepiandrosterone Sulfate	Metabolic pathways	$C_{19}H_{28}O_5S$	Steroids and steroid derivatives
pos_6051	6-Hydroxy-2-Aminopurine	Metabolic pathways; Nucleotide metabolism; Purine metabolism	$C_5H_5N_5O$	Imidazopyrimidines

Table S6. Metabolites List (Metabolites)-amino acid metabolism

ID	Metabolite	KEGG Pathway Description	Formula	HMDB Class
pos_227	4-Hydroxyproline	Metabolic pathways; D-Amino acid metabolism; Arginine and proline metabolism; ABC transporters	C5H9NO3	Carboxylic acids and derivatives
pos_298	Imidazoleacetic Acid Riboside	Histidine metabolism	C10H14N2O6	Imidazole ribonucleosides and ribonucleotides
pos_566	L-Dopa	Metabolic pathways; Biosynthesis of secondary metabolites; Tyrosine metabolism	C9H11NO4	Carboxylic acids and derivatives
pos_606	N2-Succinyl-L-Ornithine	Metabolic pathways; Arginine and proline metabolism	C9H16N2O5	Carboxylic acids and derivatives
pos_5270	Tyramine	Metabolic pathways; Methane metabolism; Biosynthesis of secondary metabolites; Tyrosine metabolism; Biosynthesis of cofactors	C8H11NO	Benzene and substituted derivatives
pos_6092	5-Hydroxy-L-Tryptophan	Metabolic pathways; Tryptophan metabolism	C11H12N2O3	Indoles and derivatives
pos_6523	Methionine Sulfoxide	Metabolic pathways; Cysteine and methionine metabolism	C5H11NO3S	Carboxylic acids and derivatives

neg_876	L-Glutathione	Metabolic pathways; Cysteine and methionine metabolism; Glutathione metabolism; ABC transporters; Biosynthesis of cofactors	$C_{10}H_{17}N_3O_6S$	Carboxylic acids and derivatives
neg_2338	4-Hydroxyphenylpyruvic Acid	Metabolic pathways; Biosynthesis of secondary metabolites; Biosynthesis of cofactors; 2-Oxocarboxylic acid metabolism; Ubiquinone and other terpenoid-quinone biosynthesis; Biosynthesis of various antibiotics; Biosynthesis of amino acids; Tyrosine metabolism; Monobactam biosynthesis; Phenylalanine, tyrosine and tryptophan biosynthesis	$C_9H_8O_4$	Benzene and substituted derivatives
neg_2555	Phenylpyruvic Acid	Metabolic pathways; Biosynthesis of secondary metabolites; 2-Oxocarboxylic acid metabolism; D-Amino acid metabolism; Biosynthesis of amino acids; Phenylalanine metabolism; Phenylalanine, tyrosine and tryptophan biosynthesis	$C_9H_8O_3$	Benzene and substituted derivatives
neg_2749	Indole-3-Acetaldehyde	Metabolic pathways; Tryptophan metabolism	$C_{10}H_9NO$	Indoles and derivatives
neg_3045	3-Hydroxycinnamic Acid	Metabolic pathways; Phenylalanine metabolism	$C_9H_8O_3$	Cinnamic acids and derivatives
neg_6949	N-Acetyl-L-Phenylalanine	Metabolic pathways; Phenylalanine metabolism	$C_{11}H_{13}NO_3$	Carboxylic acids and derivatives

neg_6955	D-3-Phenyllactic Acid	Metabolic pathways; Phenylalanine metabolism; Biosynthesis of secondary metabolites	$C_9H_{10}O_3$	Phenylpropanoic acids
neg_7116	2-Hydroxyphenylacetic Acid	Metabolic pathways; Phenylalanine metabolism	$C_8H_8O_3$	Benzene and substituted derivatives
neg_7194	Ketoleucine	Metabolic pathways; Biosynthesis of secondary metabolites; Valine, leucine and isoleucine degradation; Valine, leucine and isoleucine biosynthesis; 2-Oxocarboxylic acid metabolism; Lipoic acid metabolism; Biosynthesis of amino acids	$C_6H_{10}O_3$	Keto acids and derivatives
neg_8325	Formyl-5-Hydroxykynurenamine	Tryptophan metabolism	$C_{10}H_{12}N_2O_3$	Organooxygen compounds
neg_8428	Oxaceprol	Metabolic pathways; D-Amino acid metabolism; Arginine and proline metabolism; ABC transporters	$C_7H_{11}NO_4$	Carboxylic acids and derivatives
neg_9063	Glucosamine 6-Phosphate	Metabolic pathways; Amino sugar and nucleotide sugar metabolism; Alanine, aspartate and glutamate metabolism; Biosynthesis of nucleotide sugars	$C_6H_{14}NO_8P$	Organooxygen compounds
neg_9112	L-Saccharopine	Metabolic pathways; Lysine degradation; Biosynthesis of secondary metabolites; Lysine biosynthesis; Biosynthesis of amino acids	$C_{11}H_{20}N_2O_6$	Carboxylic acids and derivatives

Table S7. Metabolites List (Metabolites)-lipid metabolism

ID	Metabolite	KEGG Pathway Description	Formula	HMDB Class
pos_2363	Stearidonic Acid	alpha-Linolenic acid metabolism; Biosynthesis of secondary metabolites	C18H28O2	Fatty Acyls
pos_6539	Choline Glycerophosphate	Ether lipid metabolism; Glycerophospholipid metabolism	C8H20NO6P	Glycerophospholipids
neg_683	Glycerol 3-Phosphate	Metabolic pathways; Glycerophospholipid metabolism; Biosynthesis of secondary metabolites; Glycerolipid metabolism; ABC transporters	C3H9O6P	Glycerophospholipids
neg_3396	13-Hotre	alpha-Linolenic acid metabolism	C18H30O3	-
neg_3616	Coriolic Acid	Linoleic acid metabolism	C18H32O3	-
neg_3636	Galactocerebroside	Sphingolipid metabolism; Metabolic pathways	C25H47NO9	Fatty Acyls
neg_3807	Docosahexaenoic Acid	Biosynthesis of unsaturated fatty acids	C22H32O2	Fatty Acyls

neg_6086	Sphingosine 1-Phosphate	Metabolic pathways; Sphingolipid metabolism; Efferocytosis	C18H38NO5P	Sphingolipids
neg_6281	Vernolic Acid	Metabolic pathways; Linoleic acid metabolism	C18H32O3	Fatty Acyls

Table S8. Metabolites List (Metabolites)-cofactor and vitamin metabolism

ID	Metabolite	KEGG Pathway Description	Formula	HMDB Class
pos_326	Pyridoxal	Metabolic pathways; Vitamin B6 metabolism; Biosynthesis of cofactors	C8H9NO3	Pyridines and derivatives
pos_2608	Chlorophyllide A	Metabolic pathways; Biosynthesis of secondary metabolites; Porphyrin metabolism	C35H34N4O5-2	Tetrapyrroles and derivatives
pos_6190	Niacinamide	Metabolic pathways; Nicotinate and nicotinamide metabolism; Biosynthesis of cofactors	C6H6N2O	Pyridines and derivatives
neg_752	6-Lactoyltetrahydropterin	Metabolic pathways; Folate biosynthesis	C9H13N5O3	Pteridines and derivatives
neg_1973	Tetrahydrofolyl-[Glu](N)	Folate biosynthesis; Biosynthesis of cofactors	C29H37N9O12	Pteridines and derivatives
neg_2259	Pantetheine	Metabolic pathways; Biosynthesis of secondary metabolites; Pantothenate and CoA biosynthesis; Carbapenem biosynthesis	C11H22N2O4S	Carboxylic acids and derivatives

neg_2338	4-Hydroxyphenylpyruvic Acid	Metabolic pathways; Biosynthesis of secondary metabolites; Biosynthesis of cofactors; 2-Oxocarboxylic acid metabolism; Ubiquinone and other terpenoid-quinone biosynthesis; Biosynthesis of various antibiotics; Biosynthesis of amino acids; Tyrosine metabolism; Monobactam biosynthesis; Phenylalanine, tyrosine and tryptophan biosynthesis	C9H8O4	Benzene and substituted derivatives
neg_7194	Ketoleucine	Metabolic pathways; Biosynthesis of secondary metabolites; Valine, leucine and isoleucine degradation; Valine, leucine and isoleucine biosynthesis; 2-Oxocarboxylic acid metabolism; Lipoic acid metabolism; Biosynthesis of amino acids	C6H10O3	Keto acids and derivatives
neg_7224	Stercobilin	Porphyrin metabolism	C33H46N4O6	Tetrapyrroles and derivatives

Table S9. Metabolites List (Metabolites)-nucleotide metabolism

ID	Metabolite	KEGG Pathway Description	Formula	HMDB Class
pos_282	Guanosine 3'-Monophosphate	Metabolic pathways; Purine metabolism	C10H14N5O8P	Ribonucleoside 3''-phosphates
pos_6051	6-Hydroxy-2-Aminopurine	Metabolic pathways; Nucleotide metabolism; Purine metabolism	C5H5N5O	Imidazopyrimidines
pos_6086	Guanine	Metabolic pathways; Nucleotide metabolism; Purine metabolism	C5H5N5O	Imidazopyrimidines
neg_838	Cytidine 3'-Phosphate	Pyrimidine metabolism; Metabolic pathways	C9H14N3O8P	Organooxygen compounds
neg_841	Adenosine Monophosphate	Metabolic pathways; Biosynthesis of secondary metabolites; Nucleotide metabolism; Purine metabolism; Biosynthesis of cofactors	C10H14N5O7P	Purine nucleotides
neg_1466	Xanthosine	Metabolic pathways; Biosynthesis of secondary metabolites; Purine metabolism; Caffeine metabolism; ABC transporters; Nucleotide metabolism	C10H12N4O6	Purine nucleosides

Table S10. KEGG enrichment statistics (Metabolism)

Num	First Category	Second Category	Pathway Description	Pathway_ID	DA Score	P_value	P_adjust
1	Metabolism	Nucleotide metabolism	Pyrimidine metabolism	map00240	-0.1111	0.9478	1
1	Metabolism	Amino acid metabolism	Lysine degradation	map00310	-0.1111	0.9643	1
1	Metabolism	Carbohydrate metabolism	Galactose metabolism	map00052	-0.125	0.9478	1
1	Metabolism	Metabolism of cofactors and vitamins	Nicotinate and nicotinamide metabolism	map00760	-0.125	0.9478	1
1	Metabolism	Energy metabolism	Methane metabolism	map00680	-0.1429	0.924	1
1	Metabolism	Amino acid metabolism	Lysine biosynthesis	map00300	-0.1667	0.8896	1
1	Metabolism	Metabolism of other amino acids	Glutathione metabolism	map00480	-0.1667	0.8896	1
1	Metabolism	Metabolism of cofactors and vitamins	Pantothenate and CoA biosynthesis	map00770	-0.1667	0.8896	1
1	Metabolism	Biosynthesis of other secondary metabolites	Carbapenem biosynthesis	map00332	-0.3333	0.6649	1
1	Metabolism	Metabolism of cofactors and vitamins	Vitamin B6 metabolism	map00750	-0.3333	0.6649	1
1	Cellular Processes	Transport and catabolism	Efferocytosis	map04148	-0.3333	0.6649	1
1	Metabolism	Biosynthesis of other secondary metabolites	Caffeine metabolism	map00232	-0.5	0.5167	1
1	Metabolism	Lipid metabolism	Glycerolipid metabolism	map00561	-0.5	0.5167	1
1	Metabolism	Metabolism of terpenoids and polyketides	Sesquiterpenoid and triterpenoid biosynthesis	map00909	-1	0.3042	1

1	Metabolism	Amino acid metabolism	Alanine, aspartate and glutamate metabolism	map00250	0.07692	0.9923	1
1	Metabolism	Amino acid metabolism	Histidine metabolism	map00340	0.1	0.9756	1
1	Metabolism	Biosynthesis of other secondary metabolites	Biosynthesis of various plant secondary metabolites	map00999	0.1111	0.9643	1
1	Metabolism	Biosynthesis of other secondary metabolites	Biosynthesis of various antibiotics	map00998	0.125	0.9478	1
1	Metabolism	Biosynthesis of other secondary metabolites	Monobactam biosynthesis	map00261	0.2	0.8399	1
1	Metabolism	Amino acid metabolism	Valine, leucine and isoleucine biosynthesis	map00290	0.2	0.8399	1

Note:
Number: Number of metabolites enriched in this pathway;
First Category: Primary category of the metabolic pathway;
Second Category: Secondary category of the metabolic pathway;
Pathway Description: KEGG pathway name description;
Pathway ID: KEGG pathway ID;
DA Score: Differential abundance score of the metabolic pathway;
P_value_uncorrected: Uncorrected p-value, where a smaller p-value indicates greater statistical significance. Typically, a p-value less than 0.05 is considered a significant enrichment;
P_value_corrected: Corrected p-value.

Table S11. KEGG annotation statistics from the integrated analysis

Pathway ID	Pathway Description	First Category	Second Category	Metabolite number	Gene number
map00591	Linoleic acid metabolism	Metabolism	Lipid metabolism	2	1
map00600	Sphingolipid metabolism	Metabolism	Lipid metabolism	2	3
map01232	Nucleotide metabolism	Metabolism	Global and overview maps	4	8
map00760	Nicotinate and nicotinamide metabolism	Metabolism	Metabolism of cofactors and vitamins	1	6
map00998	Biosynthesis of various antibiotics	Metabolism	Biosynthesis of other secondary metabolites	1	1
map00280	Valine, leucine and isoleucine degradation	Metabolism	Amino acid metabolism	1	13
map00240	Pyrimidine metabolism	Metabolism	Nucleotide metabolism	1	7
map00380	Tryptophan metabolism	Metabolism	Amino acid metabolism	3	23

map00030	Pentose phosphate pathway	Metabolism	Carbohydrate metabolism	1	7
map00400	Phenylalanine, tyrosine and tryptophan biosynthesis	Metabolism	Amino acid metabolism	2	4
map00261	Monobactam biosynthesis	Metabolism	Biosynthesis of other secondary metabolites	1	1
map00360	Phenylalanine metabolism	Metabolism	Amino acid metabolism	5	8
map00680	Methane metabolism	Metabolism	Energy metabolism	1	10
map00052	Galactose metabolism	Metabolism	Carbohydrate metabolism	1	9
map00053	Ascorbate and aldarate metabolism	Metabolism	Carbohydrate metabolism	1	7
map00785	Lipoic acid metabolism	Metabolism	Metabolism of cofactors and vitamins	1	3
map04148	Efferocytosis	Cellular Processes	Transport and catabolism	1	3

map00130	Ubiquinone and other terpenoid-quinone biosynthesis	Metabolism	Metabolism of cofactors and vitamins	1	4
map00520	Amino sugar and nucleotide sugar metabolism	Metabolism	Carbohydrate metabolism	3	19
map00300	Lysine biosynthesis	Metabolism	Amino acid metabolism	1	9

Note: KEGG annotation statistics table, in which the first column is the KEGG ID of the pathway, the second column is the description of the pathway, the third column is the primary category of the pathway, the fourth column is the secondary category of the pathway, the fifth column is the number of metabolites annotated to the pathway, and the sixth column is the number of genes annotated to the pathway.

Table S12. Venn diagram details table

Pathway ID	Pathway Description	First Category	Second Category	Metabolite number	Metabolite list	Gene number	Gene list
map00591	Linoleic acid metabolism	Metabolism	Lipid metabolism	2	Coriolic Acid; Vernolic Acid	1	TRINITY_DN3585_c0_g1
map00600	Sphingolipid metabolism	Metabolism	Lipid metabolism	2	Galactocerebroside; Sphingosine 1-Phosphate	3	TRINITY_DN950_c0_g1; TRINITY_DN9687_c0_g1; TRINITY_DN9925_c0_g1
map01232	Nucleotide metabolism	Metabolism	Global and overview maps	4	6-Hydroxy-2-Aminopurine; Guanine; Adenosine Monophosphate; Xanthosine	8	TRINITY_DN12423_c0_g1; TRINITY_DN1852_c0_g1; TRINITY_DN2310_c0_g2; TRINITY_DN3471_c0_g1; TRINITY_DN376_c0_g1; TRINITY_DN7796_c0_g1; TRINITY_DN916_c0_g1; TRINITY_DN9972_c0_g1
map00760	Nicotinate and nicotinamide metabolism	Metabolism	Metabolism of cofactors and vitamins	1	Niacinamide	6	TRINITY_DN11344_c0_g1; TRINITY_DN1221_c0_g1; TRINITY_DN2383_c0_g1; TRINITY_DN5726_c0_g1; TRINITY_DN7443_c0_g1; TRINITY_DN942_c0_g1

map00998	Biosynthesis of various antibiotics	Metabolism	Biosynthesis of other secondary metabolites	1	4-Hydroxyphenylpyruvic Acid	1	TRINITY_DN6543_c0_g1
map00280	Valine, leucine and isoleucine degradation	Metabolism	Amino acid metabolism	1	Ketoleucine	13	TRINITY_DN10097_c0_g1; TRINITY_DN10929_c0_g1; TRINITY_DN1177_c0_g1; TRINITY_DN1593_c0_g1; TRINITY_DN2229_c0_g1; TRINITY_DN2403_c0_g1; TRINITY_DN2751_c0_g1; TRINITY_DN3341_c0_g1; TRINITY_DN3483_c0_g1; TRINITY_DN3508_c0_g1; TRINITY_DN5968_c0_g1; TRINITY_DN8405_c0_g2; TRINITY_DN9394_c0_g1
map00240	Pyrimidine metabolism	Metabolism	Nucleotide metabolism	1	Cytidine 3'-Phosphate	7	TRINITY_DN12482_c0_g1; TRINITY_DN1852_c0_g1; TRINITY_DN1931_c0_g1; TRINITY_DN2332_c0_g1; TRINITY_DN376_c0_g1; TRINITY_DN916_c0_g1; TRINITY_DN9972_c0_g1
map00380	Tryptophan metabolism	Metabolism	Amino acid metabolism	3	5-Hydroxy-L-Tryptophan; Indole-3-Acetaldehyde; Formyl-5-	23	TRINITY_DN1214_c0_g2; TRINITY_DN1539_c0_g1; TRINITY_DN1593_c0_g1; TRINITY_DN2229_c0_g1; TRINITY_DN2300_c0_g1;

					Hydroxykynurenamine		TRINITY_DN3101_c0_g1; TRINITY_DN3261_c0_g1; TRINITY_DN3422_c0_g1; TRINITY_DN3483_c0_g1; TRINITY_DN3567_c0_g1; TRINITY_DN4000_c0_g1; TRINITY_DN4077_c0_g1; TRINITY_DN4209_c0_g1; TRINITY_DN435_c0_g1; TRINITY_DN4413_c0_g1; TRINITY_DN4942_c0_g1; TRINITY_DN5968_c0_g1; TRINITY_DN8405_c0_g2; TRINITY_DN9197_c0_g1; TRINITY_DN9394_c0_g1; TRINITY_DN9419_c0_g1; TRINITY_DN9604_c0_g1; TRINITY_DN9649_c0_g1
map00030	Pentose phosphate pathway	Metabolism	Carbohydrate metabolism	1	Sedoheptulose	7	TRINITY_DN2513_c0_g1; TRINITY_DN2561_c0_g1; TRINITY_DN2802_c0_g1; TRINITY_DN3053_c0_g1; TRINITY_DN41_c0_g1; TRINITY_DN508_c0_g1; TRINITY_DN5349_c0_g1
map00400	Phenylalanine, tyrosine and tryptophan biosynthesis	Metabolism	Amino acid metabolism	2	4-Hydroxyphenylpyruvic Acid; Phenylpyruvic Acid	4	TRINITY_DN1539_c0_g1; TRINITY_DN1783_c0_g1; TRINITY_DN2009_c0_g1; TRINITY_DN3529_c0_g1

map00261	Monobactam biosynthesis	Metabolism	Biosynthesis of other secondary metabolites	1	4-Hydroxyphenylpyruvic Acid	1	TRINITY_DN9_c0_g1
map00360	Phenylalanine metabolism	Metabolism	Amino acid metabolism	5	Phenylpyruvic Acid; 3-Hydroxycinnamic Acid; N-Acetyl-L-Phenylalanine; D-3-Phenyllactic Acid; 2-Hydroxyphenylacetic Acid	8	TRINITY_DN1539_c0_g1; TRINITY_DN2693_c0_g1; TRINITY_DN3422_c0_g1; TRINITY_DN3567_c0_g1; TRINITY_DN4000_c0_g1; TRINITY_DN5190_c0_g1; TRINITY_DN8455_c0_g1; TRINITY_DN9197_c0_g1
map00680	Methane metabolism	Metabolism	Energy metabolism	1	Tyramine	10	TRINITY_DN119_c0_g1; TRINITY_DN12578_c0_g1; TRINITY_DN2352_c0_g2; TRINITY_DN2802_c0_g1; TRINITY_DN3053_c0_g1; TRINITY_DN4571_c0_g2; TRINITY_DN47_c0_g1; TRINITY_DN5994_c0_g1; TRINITY_DN9111_c0_g1; TRINITY_DN9717_c0_g1
map00052	Galactose metabolism	Metabolism	Carbohydrate metabolism	1	N-Acetyl-D-Galactosamine	9	TRINITY_DN10266_c0_g1; TRINITY_DN10974_c0_g1; TRINITY_DN1984_c0_g1; TRINITY_DN2054_c0_g2; TRINITY_DN2802_c0_g1; TRINITY_DN3834_c0_g1;

							TRINITY_DN5178_c0_g1; TRINITY_DN6457_c0_g2; TRINITY_DN9556_c0_g1
map00053	Ascorbate and aldarate metabolism	Metabolism	Carbohydrate metabolism	1	Ibuprofen Glucuronide	7	TRINITY_DN2229_c0_g1; TRINITY_DN2336_c0_g1; TRINITY_DN4250_c0_g1; TRINITY_DN5968_c0_g1; TRINITY_DN7500_c0_g1; TRINITY_DN8405_c0_g2; TRINITY_DN9394_c0_g1
map00785	Lipoic acid metabolism	Metabolism	Metabolism of cofactors and vitamins	1	Ketoleucine	3	TRINITY_DN10929_c0_g1; TRINITY_DN12486_c0_g1; TRINITY_DN4413_c0_g1
map04148	Efferocytosi s	Cellular Processes	Transport and catabolism	1	Sphingosine 1-Phosphate	3	TRINITY_DN11502_c0_g1; TRINITY_DN12297_c0_g1; TRINITY_DN4343_c0_g1
map00130	Ubiquinone and other terpenoid-quinone biosynthesis	Metabolism	Metabolism of cofactors and vitamins	1	4-Hydroxyphenylpyr uvic Acid	4	TRINITY_DN11755_c0_g1; TRINITY_DN12423_c0_g1; TRINITY_DN1539_c0_g1; TRINITY_DN3310_c0_g2
map00520	Amino sugar and nucleotide sugar metabolism	Metabolism	Carbohydrate metabolism	3	Chitobiose; N-Acetyl-D-Galactosamine; Glucosamine 6-Phosphate	19	TRINITY_DN11564_c0_g1; TRINITY_DN12390_c0_g1; TRINITY_DN1780_c1_g1; TRINITY_DN1984_c0_g1; TRINITY_DN2054_c0_g2; TRINITY_DN2240_c0_g1;

							TRINITY_DN2336_c0_g1; TRINITY_DN2375_c0_g1; TRINITY_DN2418_c0_g1; TRINITY_DN2513_c0_g1; TRINITY_DN2783_c0_g1; TRINITY_DN3834_c0_g1; TRINITY_DN5178_c0_g1; TRINITY_DN5444_c0_g2; TRINITY_DN6180_c0_g1; TRINITY_DN6180_c0_g2; TRINITY_DN706_c0_g1; TRINITY_DN9442_c0_g1; TRINITY_DN9556_c0_g1
map00300	Lysine biosynthesis	Metabolism	Amino acid metabolism	1	L-Saccharopine	9	TRINITY_DN10798_c0_g1; TRINITY_DN1428_c0_g1; TRINITY_DN1539_c0_g1; TRINITY_DN1766_c0_g1; TRINITY_DN2087_c0_g1; TRINITY_DN3914_c0_g1; TRINITY_DN4447_c0_g1; TRINITY_DN934_c0_g1; TRINITY_DN9_c0_g1

Note: The Venn diagram illustrates the pathways annotated by the gene set and those annotated by the metabolite set, with a focus on the pathways commonly annotated by both the gene set and the metabolite set.

Table S13. KEGG pathway annotation statistics table

Pathway ID	Pathway Description	First Category	Second Category	Metabolite number	Gene number
map00591	Linoleic acid metabolism	Metabolism	Lipid metabolism	2	1
map00600	Sphingolipid metabolism	Metabolism	Lipid metabolism	2	3
map01232	Nucleotide metabolism	Metabolism	Global and overview maps	4	8
map00760	Nicotinate and nicotinamide metabolism	Metabolism	Metabolism of cofactors and vitamins	1	6
map00998	Biosynthesis of various antibiotics	Metabolism	Biosynthesis of other secondary metabolites	1	1
map00280	Valine, leucine and isoleucine degradation	Metabolism	Amino acid metabolism	1	13
map00240	Pyrimidine metabolism	Metabolism	Nucleotide metabolism	1	7
map00380	Tryptophan metabolism	Metabolism	Amino acid metabolism	3	23

map00030	Pentose phosphate pathway	Metabolism	Carbohydrate metabolism	1	7
map00400	Phenylalanine, tyrosine and tryptophan biosynthesis	Metabolism	Amino acid metabolism	2	4
map00261	Monobactam biosynthesis	Metabolism	Biosynthesis of other secondary metabolites	1	1
map00360	Phenylalanine metabolism	Metabolism	Amino acid metabolism	5	8
map00680	Methane metabolism	Metabolism	Energy metabolism	1	10
map00052	Galactose metabolism	Metabolism	Carbohydrate metabolism	1	9
map00053	Ascorbate and aldarate metabolism	Metabolism	Carbohydrate metabolism	1	7
map00785	Lipoic acid metabolism	Metabolism	Metabolism of cofactors and vitamins	1	3
map04148	Efferocytosis	Cellular Processes	Transport and catabolism	1	3

map00130	Ubiquinone and other terpenoid-quinone biosynthesis	Metabolism	Metabolism of cofactors and vitamins	1	4
map00520	Amino sugar and nucleotide sugar metabolism	Metabolism	Carbohydrate metabolism	3	19
map00300	Lysine biosynthesis	Metabolism	Amino acid metabolism	1	9

Note: KEGG annotation statistics table, in which the first column is the KEGG ID of the pathway, the second column is the pathway description, the third column is the primary category of the pathway, the fourth column is the secondary category of the pathway, the fifth column is the number of metabolites annotated to the pathway, and the sixth column is the number of genes annotated to the pathway.

Figure Legend:
Green background: Represents all genes/transcripts annotated in this project.
Red border: Indicates up-regulated genes/transcripts.
Blue border: Indicates down-regulated genes/transcripts.
Red and blue combined border: Represents a set containing both up-regulated and down-regulated genes/transcripts.

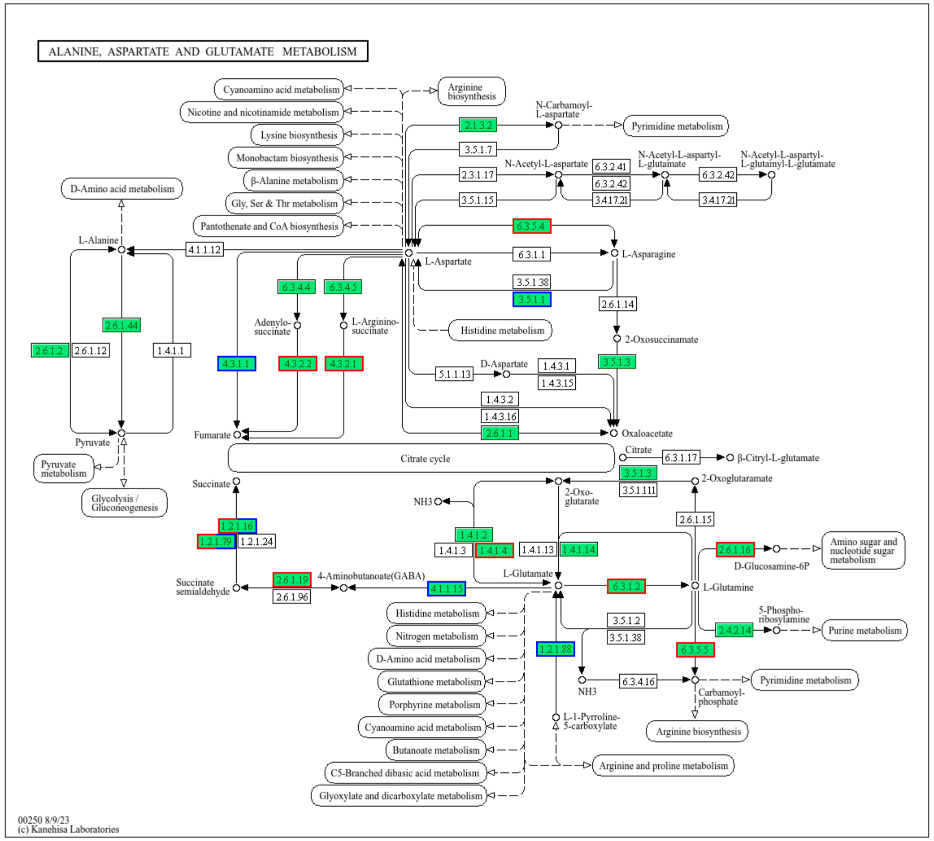

Fig. S4. KEGG enrichment of alanine, aspartate, and glutamate pathway based on transcriptomic data in *Fusarium* sp. DLT-118

4.3.2.2: *purB*; 4.3.2.1: *argH*; 6.3.5.4: *asnB*; 1.4.1.4: *E 1.4.1.4*; 2.6.1.19: *ABAT*; 6.3.1.2: *glnA*; 2.6.1.16: *glmS*; 6.3.5.5: *CAD*

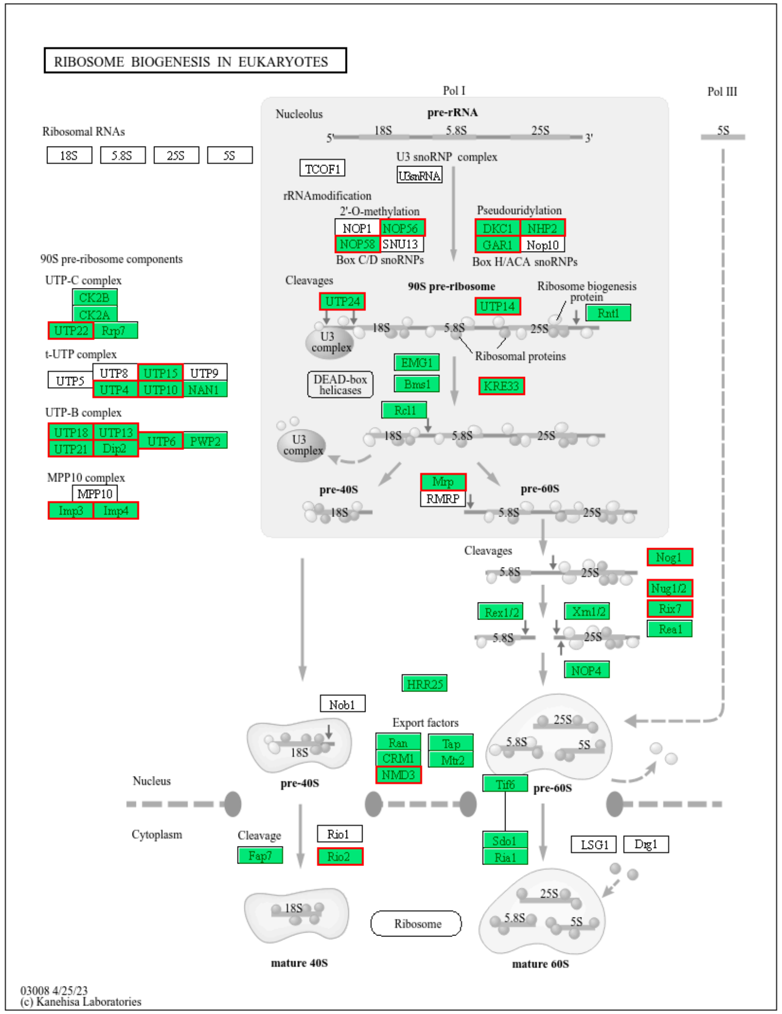

Fig. S5. KEGG enrichment of ribosome biogenesis in eukaryotes pathway based on transcriptomic data in *Fusarium* sp. DLT-118

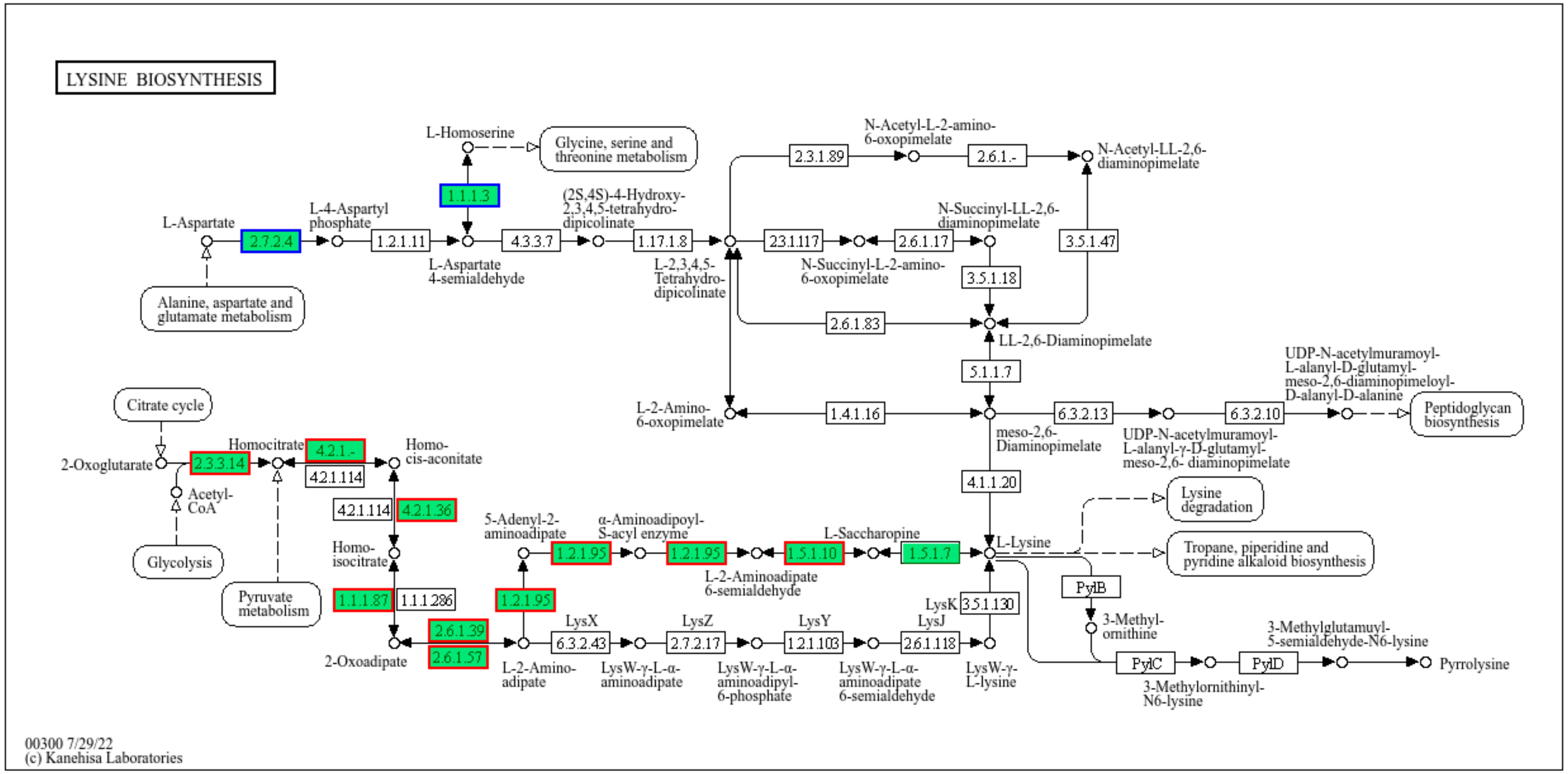

Fig. S6. KEGG enrichment of lysine biosynthesis pathway based on transcriptomic data in *Fusarium* sp. DLT-118

2.3.3.14: *LYS21*; 4.2.1.-: *ACO2*; 4.2.1.36: *LYS4*; 1.1.1.87: *LYS12*; 2.6.1.39: *AADAT*; 2.6.1.57: *ARO8*; 1.2.1.95: *LYS2*; 1.5.1.10: *LYS9*

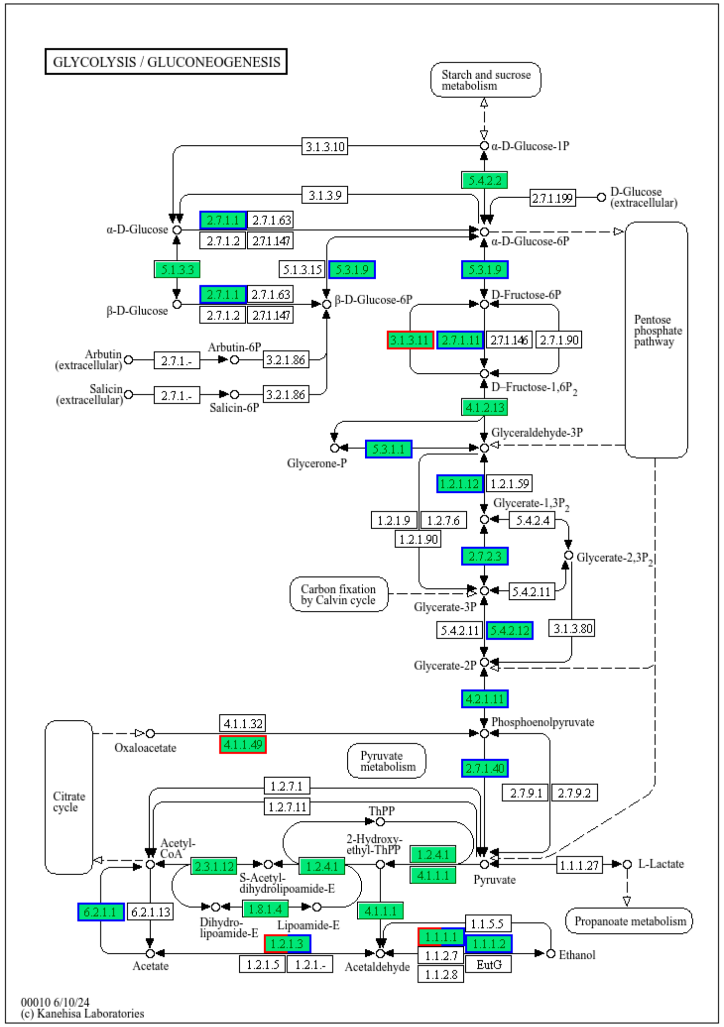

Fig. S7. KEGG enrichment of glycolysis/gluconeogenesis pathway based on transcriptomic data in *Fusarium* sp. DLT-118

3.1.3.11: *FBP*, *glpx*, *glpx-SEBP*, *fbp-SEBP*, *fbp3*; 4.1.1.49: *pckA*

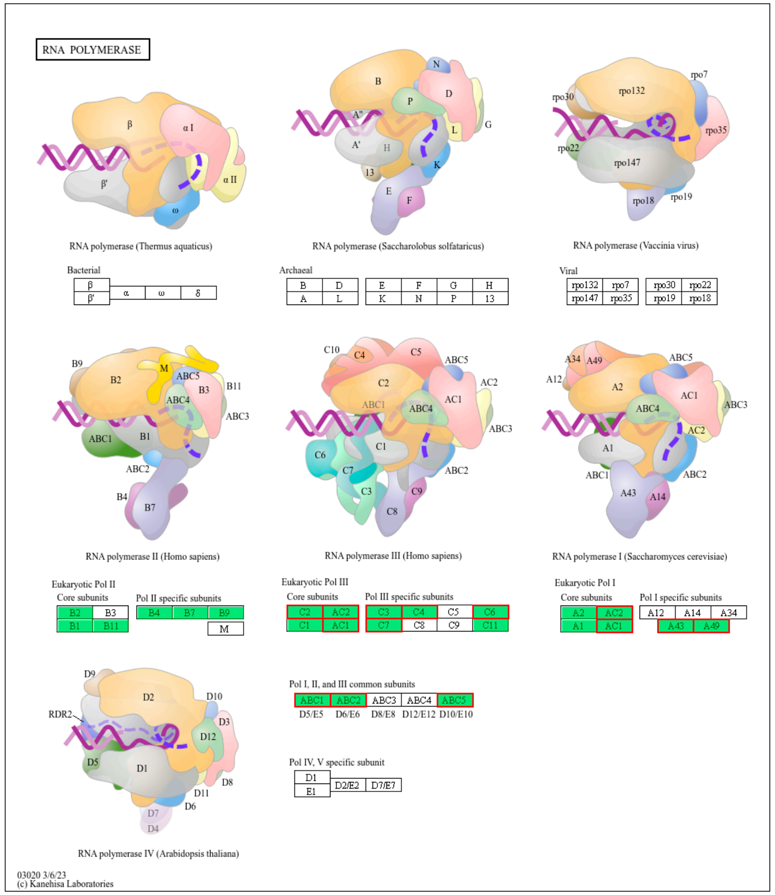

Fig. S8. KEGG enrichment of RNA polymerase pathway based on transcriptomic data in *Fusarium* sp. DLT-118

C2: *RPC2*; AC1: *RPAC1*; AC2: *RPAC2*; C3: *RPC3*; C4: *RPC4*; C7: *RPC7*; C6: *RPC6*; AC43: *RPAC43*; AC49: *RPAC49*; ABC1: *RPABC1*; ABC2: *RPABC2*; ABC5: *RPABC5*

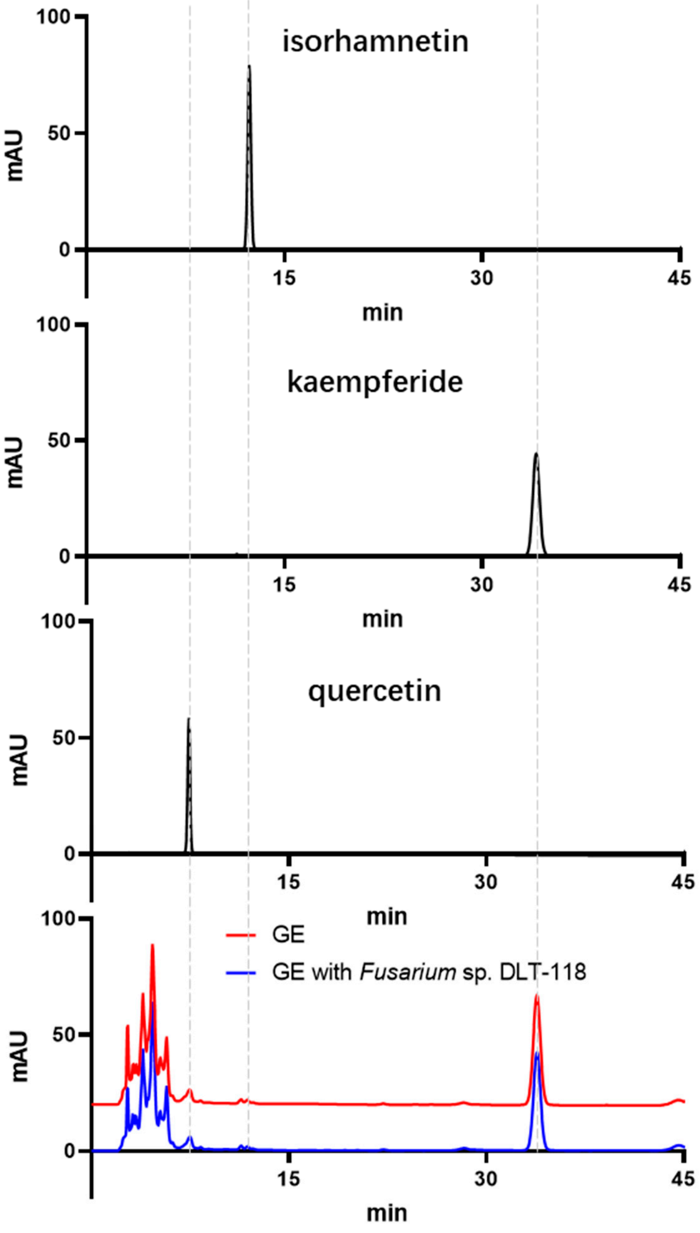

Fig. S9. Effect of *Fusarium* sp. DLT-118 on the flavonoids content in GE (360 nm)

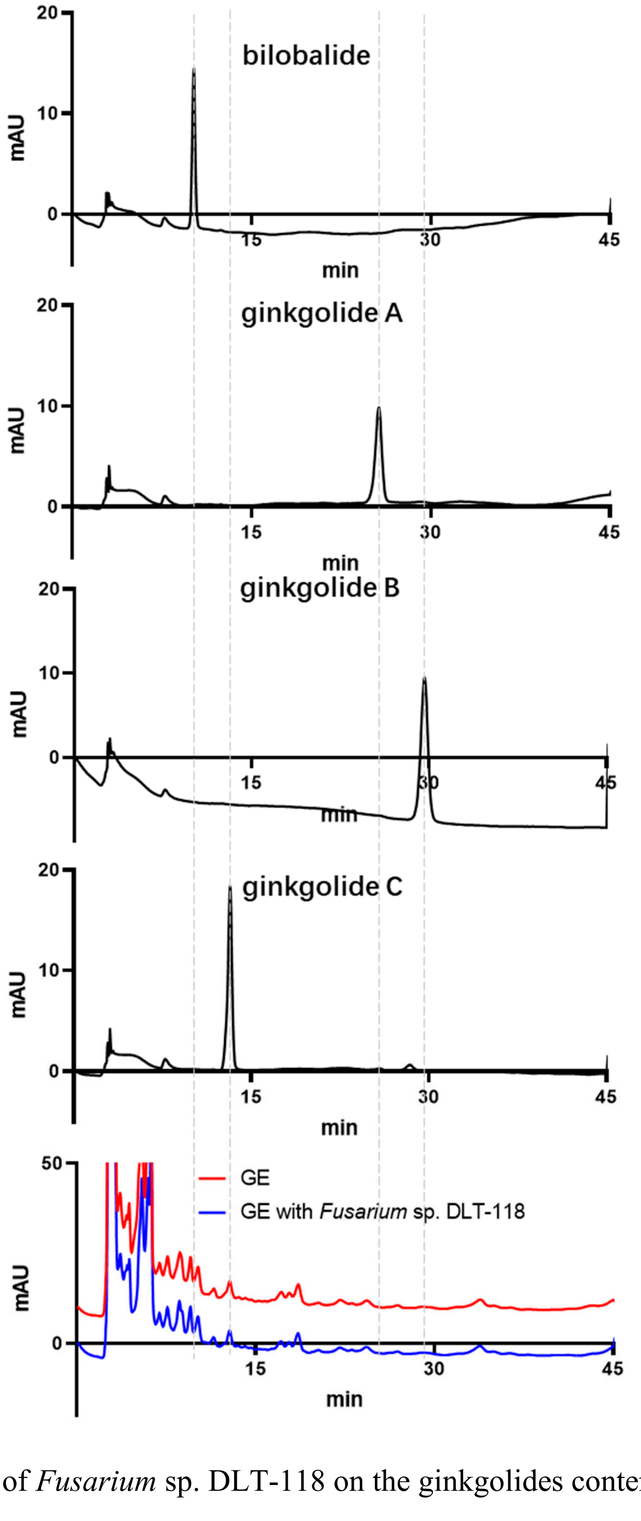

Fig. S10. Effect of *Fusarium* sp. DLT-118 on the ginkgolides content in GE (222 nm)

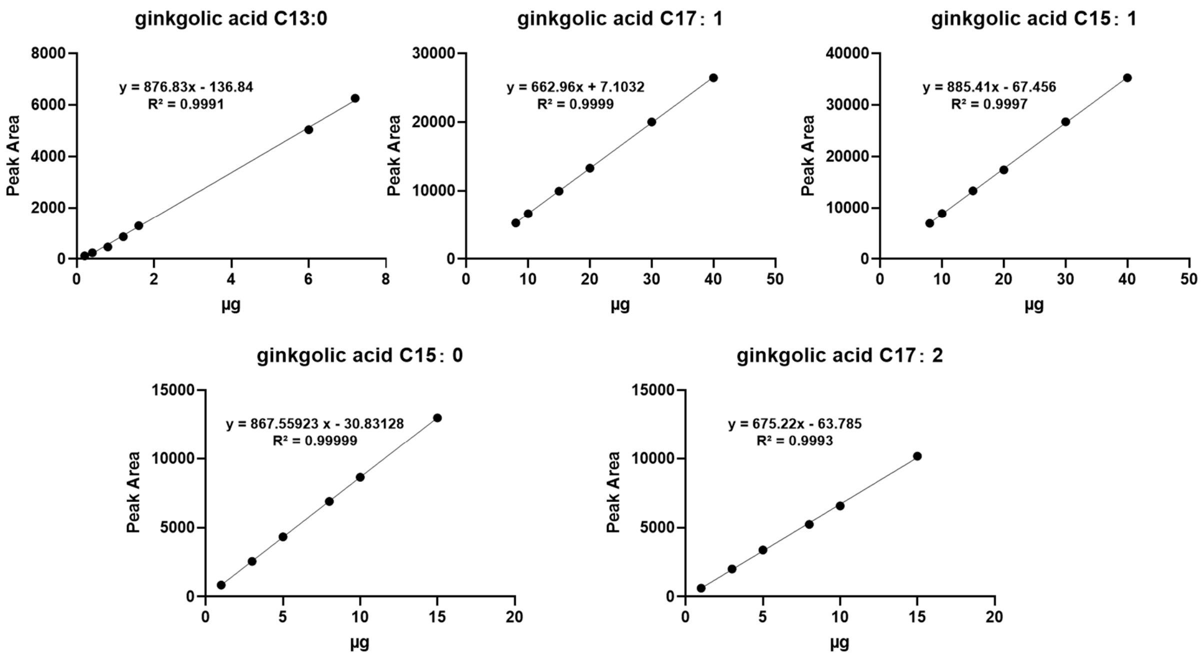

Fig. S11. Standard curves for the quantification of ginkgolic acids

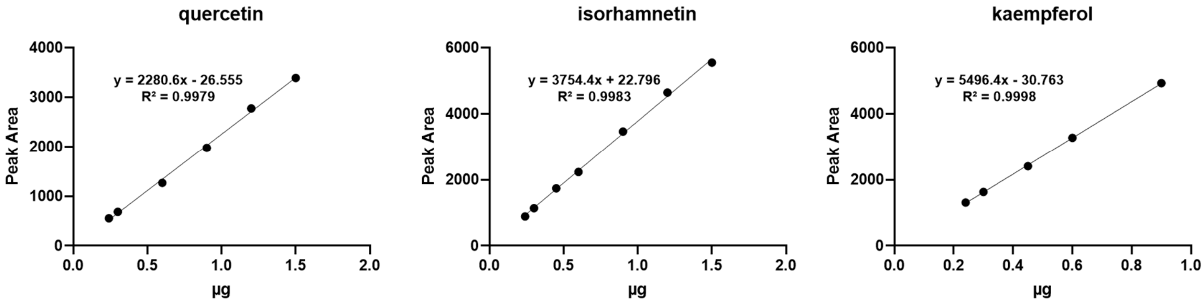

Fig. S12. Standard curves for the quantification of flavonoids

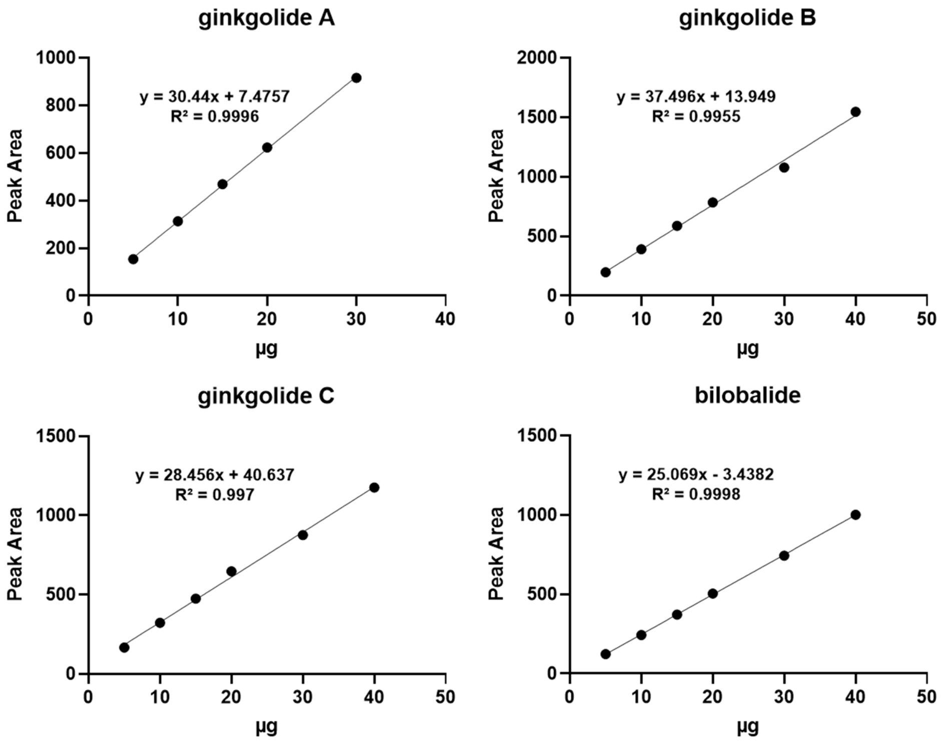

Fig. S13. Standard curves for the quantification of ginkgolides

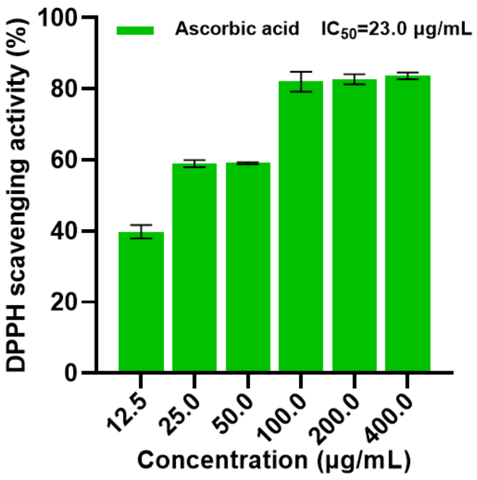

Fig. S14. DPPH scavenging activity of Ascorbic acid

Table S14 Identification results of metabolites from the fermentation products of *Fusarium* sp. DLT-118 by non-targeted metabolomics

Title	RT (min)	theoretical_mz	Precursor m/z	PPM	Adduct	Formula	Superclass	Dot product	Reverse dot product	Fragment presence %
Norharman	2.10673	169.076024	169.0736	14.33674594	[M+H]+	C11H8N2	Organoheterocyclic compounds	94.9	96.7	92.3
N-Lauryldiethanolamine	1.80648	274.274055	274.2729	4.211116505	[M+H]+	C16H35NO2	Organic nitrogen compounds	93.8	94.9	90
Acuminatum B	1.28157	888.544084	888.5442	0.130550641	[M+H]+	C45H73N7O11	Organic acids and derivatives	92.6	86.8	87.8
Cyclo(glycylprolyl)	1.42822	155.081504	155.0802	8.408481775	[M+H]+	C7H10N2O2	Organic acids and derivatives	92.1	97.4	88.9
Harmane	2.12615	183.091674	183.0925	4.511401212	[M+H]+	C12H10N2	Alkaloids and derivatives	91.7	95.3	94.1
Betaine	1.46197	118.086255	118.0858	3.853115674	[M+H]+	C5H11NO2	Organic acids and derivatives	91.3	93.6	100
(Quinolin-4-yl)methanol	1.73828	160.07569	160.0741	9.932801164	[M+H]+	C10H9NO	Organoheterocyclic compounds	90.7	96.7	100
Acuminatum C	1.28157	874.528434	874.5226	6.671023803	[M+H]+	C44H71N7O11	Organic acids and derivatives	89.7	83.4	77.2
N-Acetyltyramine	1.36237	180.101905	180.1013	3.359209332	[M+H]+	C10H13NO2	Benzenoids	87.5	79.2	100
Isoleucine	1.51322	132.101905	132.1018	0.794840922	[M+H]+	C6H13NO2	Organic acids and derivatives	87	93.7	61.5
Stachydrine	1.4789	144.101905	144.1006	9.056091243	[M+H]+	C7H13NO2	Organic acids and derivatives	86.9	92.6	100
ISOGINKGETIN	3.2716	567.128576	567.1258	4.894833583	[M+H]+	C32H22O10	Phenylpropanoids and polyketides	86.5	80.8	48.1
Phthalic anhydride	52.8689	149.023321	149.0224	6.180240742	[M+H]+	C8H4O3	Organoheterocyclic compounds	85.6	87.5	80
2,3,4,9-Tetrahydro-1H-.beta.-carboline-3-carboxylic acid	1.75532	217.097154	217.0978	2.975626295	[M+H]+	C12H12N2O2	Organoheterocyclic compounds	85	86.5	92.3
Nicotinamide	1.61652	123.055289	123.0541	9.662323413	[M+H]+	C6H6N2O	Organoheterocyclic compounds	84.3	86.3	58.8
Tetrahydroharman-3-carboxylic acid	1.77233	231.112804	231.1152	10.36723175	[M+H]+	C13H14N2O2	Alkaloids and derivatives	83	90.9	84.8

Adenosine	1.58227	268.104031	268.1039	0.488616301	[M+H]+	C10H13N5O4	Nucleosides, nucleotides, and analogues	82.6	86.4	100
Phenylacetaldehyde	1.36237	121.064791	121.0634	11.48971545	[M+H]+	C8H8O	Benzenoids	82.6	85.2	92.3
Cinobufagin	1.36237	443.242816	443.2376	11.76781622	[M+H]+	C26H34O6	Lipids and lipid-like molecules	80.7	82.5	75
Indoline	1.63387	120.080775	120.0799	7.286761765	[M+H]+	C8H9N	Organoheterocyclic compounds	79.4	48.1	48.1
Arginine	1.68642	175.118952	175.1201	6.555544028	[M+H]+	C6H14N4O2	Organic acids and derivatives	79	89.5	88.9
1-Monostearin	53.0674	359.315586	359.3161	1.430497368	[M+H]+	C21H42O4	Lipids and lipid-like molecules	78.1	78.7	61.5
Betamethasone 9,11-epoxide	1.42822	373.200951	373.1978	8.443172483	[M+H]+	C22H28O5	Lipids and lipid-like molecules	77.9	42.8	57.4
3-Indoleacrylic acid	1.73828	188.070605	188.0704	1.090016167	[M+H]+	C11H9NO2	Organoheterocyclic compounds	76.1	81.3	85.7
1-(p-Tolyl)cyclopropanecarbonitrile	1.721	158.096425	158.0971	4.26954626	[M+H]+	C11H11N	Benzenoids	74.4	76.4	72.7
6-Isoquinolinol	1.73828	146.06004	146.0602	1.09543993	[M+H]+	C9H7NO	Organoheterocyclic compounds	73.4	88.3	87.5
(9S,18R,21R)-15-benzyl-6-(4-((tert-butoxycarbonyl)oxy)benzyl)-21-(3-guanidinopropyl)-18-isobutyl-2,2,9-trimethyl-4,7,10,13,16,19-hexaoxo-3-oxa-5,8,11,14,17,20-hexaazadocosan-22-oic acid	1.44488	926.498197	926.4851	14.13602319	[M+H]+	C45H67N9O12	Organic acids and derivatives	73.3	75	100
CUDC 101	1.41147	435.202682	435.209	14.51737377	[M+H]+	C24H26N4O4	Organoheterocyclic compounds	71.3	76.6	100
Indarubicin	2.2045	263.081504	263.0817	0.745016267	[M+H]+	C16H10N2O2	Organoheterocyclic compounds	70.9	83	84.6
N-Cyclohexyl-1-pyrrolidinecarboxamide	1.75532	197.164839	197.1659	5.381284033	[M+H]+	C11H20N2O	Organoheterocyclic compounds	65.8	72.4	66.7
Solanidine	2.62618	398.34174	398.3432	3.665194614	[M+H]+	C27H43NO	Lipids and lipid-like molecules	65	68.5	66.7

Eplerenone	1.41147	415.211516	415.2072	10.39470206	[M+H]+	C24H30O6	Lipids and lipid-like molecules	64.9	84.7	100
Tubastatin A	3.38532	336.170653	336.1745	11.44359261	[M+H]+	C20H21N3O2	Organoheterocyclic compounds	64.9	75	100
N-Fructosyl phenylalanine	1.51322	328.13908	328.1394	0.975196249	[M+H]+	C15H21NO7	Organic acids and derivatives	64.6	65.2	53.5
Phenoxyacetyl fentanyl	1.33012	415.238004	415.244	14.43991143	[M+H]+	C27H30N2O2	Benzenoids	64.5	82.4	50
Demissidine	2.8646	400.35739	400.3591	4.271183804	[M+H]+	C27H45NO	Lipids and lipid-like molecules	64.3	73.9	50
Thiocarlide	1.36237	401.225725	401.2272	3.676234867	[M+H]+	C23H32N2O2S	Benzenoids	62.4	84.8	100
Cediranib	1.41147	451.213995	451.2073	14.83774899	[M+H]+	C25H27FN4O3	Organic oxygen compounds	60	84.4	50
N-Fructosyl isoleucine	1.4789	294.15473	294.1533	4.861387066	[M+H]+	C12H23NO7	Organic acids and derivatives	59.5	71.6	73.5
Tetrahydrocurcumin	1.46197	373.164566	373.1595	13.57577986	[M+H]+	C21H24O6	Phenylpropanoids and polyketides	58.3	77.9	50
Helenalin tiglate	1.3784	345.169651	345.165	13.47453343	[M+H]+	C20H24O5	Lipids and lipid-like molecules	57.2	86.9	100
2-Ethylisonicotinonitrile	1.77233	133.076024	133.0753	5.440499184	[M+H]+	C8H8N2	Organoheterocyclic compounds	57.2	85	66.7
4-Aminophenol	1.63387	110.06004	110.0595	4.9064129	[M+H]+	C6H7NO	Benzenoids	55.1	79.3	83.3
Cyclo(prolylvalyl)	1.36237	197.128454	197.1284	0.273933057	[M+H]+	C10H16N2O2	Organic acids and derivatives	54.5	80.5	50
Linamarin	1.56497	248.112865	248.1117	4.695443745	[M+H]+	C10H17NO6	Organic oxygen compounds	53.5	70.1	63
Imidocarb	1.51322	349.177135	349.1738	9.551026301	[M+H]+	C19H20N6O	Benzenoids	53.5	72.5	50
2,2'-(Hexadecylazanediyl)bis(ethan-1-ol)	2.10673	330.336655	330.3382	4.677046814	[M+H]+	C20H43NO2	Organic nitrogen compounds	52.7	86.1	50
Formoterol	1.41147	345.180884	345.1808	0.243350672	[M+H]+	C19H24N2O4	Benzenoids	51.8	83.1	66.7
Alkaloid SN-f	2.12615	900.49513	900.5086	14.95843737	[M+H]+	C45H73NO17	Lipids and lipid-like molecules	49.5	63	50
D-ribo-Phytosphingosine	1.77233	318.30027	318.2993	3.047436937	[M+H]+	C18H39NO3	Organic nitrogen compounds	49.3	80.3	50
Benzyl carfentanil	1.78957	381.217269	381.2155	4.640398386	[M+H]+	C23H28N2O3	Organoheterocyclic compounds	48.5	69.9	50

Resmethrin	1.34618	339.195471	339.1926	8.464146032	[M+H]+	C22H26O3	Lipids and lipid-like molecules	48.3	52.5	66.7
p-Fluoro tetrahydrofuran fentanyl	1.36237	397.228582	397.23	3.569733056	[M+H]+	C24H29FN2O2	Organoheterocyclic compounds	47.8	75	100
Demethylcitalopram	1.42822	311.155417	311.1583	9.265466203	[M+H]+	C19H19FN2O	Benzenoids	46.4	77.1	42.9
Amorfrutin A	1.36237	341.174736	341.1708	11.53661038	[M+H]+	C21H24O4	Phenylpropanoids and polyketides	46.2	79.2	75
.beta.-Hydroxythiofentanyl	1.3784	359.178775	359.1799	3.132144988	[M+H]+	C20H26N2O2S	Benzenoids	46	74.8	100
Bicifadine	2.18072	174.127725	174.1268	5.31219253	[M+H]+	C12H15N	Organoheterocyclic compounds	44.7	48.1	57.1
L-Tryptophane	1.721	205.097154	205.0998	12.90120291	[M+H]+	C11H12N2O2	Organoheterocyclic compounds	44.6	83.8	100
Speciosine	1.5306	478.222415	478.2261	7.70561957	[M+H]+	C28H31NO6	Hydrocarbon derivatives	44.6	70	50
6.alpha.-Hydroxybudesonide	1.39503	447.237731	447.2328	11.02545617	[M+H]+	C25H34O7	Lipids and lipid-like molecules	44.6	74	100
Bergamottin	1.44488	339.159086	339.1595	1.220666104	[M+H]+	C21H22O4	Lipids and lipid-like molecules	43.8	78.4	50
2-Fluorobutyrylfentanyl	1.39503	369.233667	369.2287	13.45218609	[M+H]+	C23H29FN2O	Organoheterocyclic compounds	43.5	75	100
2-(3-Aminophenyl)-4-quinolinecarboxylic acid	2.3732	265.097154	265.0972	0.173521289	[M+H]+	C16H12N2O2	Organoheterocyclic compounds	43.4	68.5	60
p-Fluoroacetyl fentanyl	1.36237	341.202367	341.2047	6.837584453	[M+H]+	C21H25FN2O	Benzenoids	42.3	82.7	50
Imiprothrin	1.59938	319.165234	319.1606	14.51912523	[M+H]+	C17H22N2O4	Organoheterocyclic compounds	42.2	56.1	41.7
Lotaustralin	1.46197	262.128515	262.1281	1.583192885	[M+H]+	C11H19NO6	Organic oxygen compounds	42.1	51.3	44.9
Menisdaurin D	1.51322	316.13908	316.1379	3.732534427	[M+H]+	C14H21NO7	Organic oxygen compounds	41.9	82.5	100
Pimozide	1.5306	462.235144	462.2306	9.830494412	[M+H]+	C28H29F2N3O	Benzenoids	41.8	71.6	50
1H-Indole-3-carboxylic acid, 1-(cyclohexylmethyl)-, 8-quinolinyl ester	2.54697	385.191054	385.1897	3.51513875	[M+H]+	C25H24N2O2	Organoheterocyclic compounds	41.3	82.2	50
Gibberellin A4	52.479	333.169651	333.1659	11.25852847	[M+H]+	C19H24O5	Lipids and lipid-like molecules	39.4	87.9	100

2,2'-(Tetradecylimino)diethanol	1.92358	302.305355	302.3061	2.464395644	[M+H]+	C18H39NO2	Organic nitrogen compounds	38.9	85.5	53.8
2-Imino-1-imidazolidineacetic acid	1.77233	144.076753	144.0789	14.90177947	[M+H]+	C5H9N3O2	Organic acids and derivatives	37.7	86.3	100
dG-C8-IQ	1.54777	464.178927	464.1834	9.636370244	[M+H]+	C21H21N9O4	Nucleosides, nucleotides, and analogues	35.1	71.5	50
CUMYL-PeGACLONE	1.46197	373.227439	373.2304	7.933500302	[M+H]+	C25H28N2O	Organoheterocyclic compounds	34.9	75.7	100
Dibutyl phthalate	52.9169	279.159086	279.1596	1.841244028	[M+H]+	C16H22O4	Benzenoids	33.3	84.7	100
2,3',4,5'-Tetramethoxystilbene	52.8849	301.143436	301.1404	10.08157455	[M+H]+	C18H20O4	Phenylpropanoids and polyketides	31.9	73.2	50
2-Pyridinylmethyl 2-(4-isobutylphenyl)propanoate	1.77233	298.180155	298.1794	2.532026318	[M+H]+	C19H23NO2	Lipids and lipid-like molecules	30.5	52.1	50
3-Cyclohexyl-1-(piperazin-1-yl)propan-1-one	2.2008	225.196139	225.1944	7.722157261	[M+H]+	C13H24N2O	Lipids and lipid-like molecules	30.3	87.8	100
Aniquinazoline B	1.46197	474.213581	474.2171	7.420706916	[M+H]+	C26H27N5O4	Organoheterocyclic compounds	29.2	87.9	100
DMCM	1.90682	315.133934	315.1376	11.63314897	[M+H]+	C17H18N2O4	Organoheterocyclic compounds	28.9	71.9	82.6
Alprenolol	52.5588	250.180155	250.1767	13.8100482	[M+H]+	C15H23NO2	Benzenoids	28.8	74.5	50
Fluvastatin	1.73828	412.191863	412.1962	10.52179917	[M+H]+	C24H26FNO4	Organoheterocyclic compounds	28.5	75	100
Pentobarbital	1.34618	227.139019	227.138	4.486239328	[M+H]+	C11H18N2O3	Organoheterocyclic compounds	27.9	74.3	50
Erianin	1.56497	319.154001	319.1559	5.950105573	[M+H]+	C18H22O5	Phenylpropanoids and polyketides	27.1	67.6	75
Aminoglutethimide	1.66888	233.128454	233.1273	4.950060708	[M+H]+	C13H16N2O2	Benzenoids	26.7	80.8	42.9
Butyrylfentanyl	1.90682	351.243089	351.2463	9.14181688	[M+H]+	C23H30N2O	Organoheterocyclic compounds	26.5	81.5	50
Taprostene (free acid)	1.3784	399.216601	399.212	11.52507182	[M+H]+	C24H30O5	Benzenoids	26.3	86.1	66.7
Lofepramine	2.36575	419.188467	419.1865	4.692400089	[M+H]+	C26H27ClN2O	Organoheterocyclic compounds	25.7	73	50
MMB022	1.75532	343.201619	343.1988	8.213830716	[M+H]+	C20H26N2O3	Organic acids and derivatives	23.4	74	50

Ethyl 3-tert-butyl-1-(4-fluorobenzyl)-1H-pyrazole-5-carboxylate	2.23638	305.165982	305.1685	8.251247349	[M+H]+	C17H21FN2O2	Organoheterocyclic compounds	22.3	74.6	100
Pantothenate	1.33012	220.11795	220.1161	8.404584906	[M+H]+	C9H17NO5	Organic oxygen compounds	20.7	77.4	66.7
6-ethyl-3-(4-isopropoxyphenyl)-4-oxo-4H-chromen-7-yl acetate	1.68642	367.154001	367.1516	6.539490223	[M+H]+	C22H22O5	Phenylpropanoids and polyketides	20.2	80.4	100
Tanshinone IIA	1.721	295.132871	295.1319	3.290043555	[M+H]+	C19H18O3	Lipids and lipid-like molecules	19.8	66.8	100
(24E)-3-Oxodammara-1,12,24-trien-26-oic acid	1.36237	453.336321	453.3406	9.438908382	[M+H]+	C30H44O3	Lipids and lipid-like molecules	19.4	81.5	75
1-Cyclobutyl-4-piperidinamine	1.77233	155.154274	155.152	14.65638001	[M+H]+	C9H18N2	Organoheterocyclic compounds	19	80.3	50
1-Isothiocyanato-8-(methylsulfinyl)-octane	1.46197	234.098082	234.0956	10.60239357	[M+H]+	C10H19NOS2	Organosulfur compounds	18.3	72.4	58.3
Triamterene	1.54777	254.114869	254.1151	0.909037716	[M+H]+	C12H11N7	Organoheterocyclic compounds	18.3	87.1	66.7
Flavaspidic acid AB	2.43943	419.170046	419.1719	4.423025972	[M+H]+	C22H26O8	Organic oxygen compounds	17.5	83.7	75
Ritalinic acid	1.84022	220.133205	220.1312	9.108121603	[M+H]+	C13H17NO2	Organic nitrogen compounds	17	88	100
N-Butyl-2-(1H-indol-3-yl)-2-oxoacetamide	1.721	245.128454	245.1291	2.635352973	[M+H]+	C14H16N2O2	Organoheterocyclic compounds	16.9	87.7	100
2,2'-((4-Fluoro-3-nitrophenyl)azanediyl)diethanol	1.42822	245.093212	245.0921	4.537049357	[M+H]+	C10H13FN2O4	Benzenoids	16.9	87	100
Norfentanyl	1.721	233.164839	233.1658	4.121547675	[M+H]+	C14H20N2O	Benzenoids	16.8	74.4	50
Pyridoxal	1.3139	168.06552	168.0658	1.66601692	[M+H]+	C8H9NO3	Organoheterocyclic compounds	16.1	87.7	100
Metaraminol	1.46197	168.101905	168.101	5.383639168	[M+H]+	C9H13NO2	Benzenoids	16.1	87.6	100
Phenylalanylphenylalanine	1.78957	313.154669	313.1547	0.098992616	[M+H]+	C18H20N2O3	Organic acids and derivatives	14.6	84.7	80
1-Benzyl-4-(2-pyridinyl)piperazine	1.63387	254.165173	254.1622	11.69711792	[M+H]+	C16H19N3	Organoheterocyclic compounds	14.4	87.9	100
Ketotifen	1.51322	310.126011	310.1282	7.058421165	[M+H]+	C19H19NOS	Organoheterocyclic compounds	14.3	84.4	50

8-(2-Hydroxybenzamido)octanoic acid	1.4789	280.154335	280.1506	13.3319372	[M+H]+	C15H21NO4	Benzenoids	13.4	88.3	66.7
Octamethylpyrophosphoramide	1.90682	287.139639	287.1414	6.132904555	[M+H]+	C8H24N4O3P2	Organic acids and derivatives	13.4	77.6	50
N-(1-Benzyl-4-piperidinyl)-2,2,2-trifluoroacetamide	1.90682	287.136573	287.1388	7.755891131	[M+H]+	C14H17F3N2O	Organoheterocyclic compounds	13.4	84.9	50
4-(1H-Indol-3-yl)-1-(morpholin-4-yl)butan-1-one	1.77233	273.159754	273.159	2.760289497	[M+H]+	C16H20N2O2	Organoheterocyclic compounds	12.8	87.2	100
4-(Adamantyl-1-sulfinyl)morpholine	1.721	270.152226	270.1545	8.417476449	[M+H]+	C14H23NO2S	Organoheterocyclic compounds	12.3	75.6	60
Tetrahydrocoptisine	1.66888	324.123035	324.1207	7.204054473	[M+H]+	C19H17NO4	Alkaloids and derivatives	11.5	70	100
Sotalol	1.7037	273.12674	273.1241	9.665842312	[M+H]+	C12H20N2O3S	Benzenoids	11.5	87.4	100
1-Tetrahydro-3-furanyl-4-piperidinamine	1.75532	171.149189	171.1491	0.52001415	[M+H]+	C9H18N2O	Organoheterocyclic compounds	10.8	80.1	50
Cnicin	1.68642	379.175131	379.1768	4.401659981	[M+H]+	C20H26O7	Lipids and lipid-like molecules	10.1	79.6	80
Norclozapine	1.78957	313.12145	313.1198	5.26952082	[M+H]+	C17H17ClN4	Organoheterocyclic compounds	8.5	75	100
Desmedipham	1.97303	301.118284	301.1179	1.275246375	[M+H]+	C16H16N2O4	Benzenoids	2.1	84.6	100

Note:

The three metrics, Dot product, Reverse dot product, and Fragment presence %, represent the secondary spectrum matching procedure, where positive values for these columns in the identification results table indicate the highest-confidence identification results.

Table S15 Annotation details of differentially expressed unigenes

	gene_id	nr_description	fc	log2fc	pvalue	padjust	regulate	go	paths
glnA	TRINITY_DN1265_c0_g1	glutamate--ammonia ligase [Fusarium torreyae]	2.231557	1.15805	9.44E-14	2.30E-12	up	GO:0004356 (molecular_function:glutamate-ammonia ligase activity); GO:0005737 (cellular_component:cytoplasm); GO:0005524(molecular_function:ATP binding); GO:0006542 (biological_process:glutamine biosynthetic process)	map00630 (Glyoxylate and dicarboxylate metabolism); map00220 (Arginine biosynthesis); map00250 (Alanine, aspartate and glutamate metabolism); map00910 (Nitrogen metabolism)
	TRINITY_DN1571_c0_g1	hypothetical protein NW762_006288 [Fusarium torreyae]	3.805495	1.928084	6.74E-13	1.48E-11	up	GO:0004356 (molecular_function: glutamate-ammonia ligase activity); GO:0016020 (cellular_component:membrane); GO:0006542 (biological_process:glutamine biosynthetic process)	map00630 (Glyoxylate and dicarboxylate metabolism); map00220 (Arginine biosynthesis); map00250 (Alanine, aspartate and glutamate metabolism); map00910 (Nitrogen metabolism)
asnB	TRINITY_DN65_c0_g1	asparagine synthetase [Fusarium torreyae]	3.799618	1.925854	3.29E-08	3.46E-07	up	GO:0005829 (cellular_component:cytosol); GO:0006529 (biological_process:asparagine biosynthetic process); GO:0006541 (biological_process:glutamine metabolic process); GO:0005524 (molecular_function:ATP binding); GO:0004066 (molecular_function:asparagine synthase (glutamine-hydrolyzing) activity)	map00250 (Alanine, aspartate and glutamate metabolism)
NOP56	TRINITY_DN4614_c0_g1	hypothetical protein LZL87_007343 [Fusarium oxysporum]	3.307042	1.725541	7.65E-05	0.000398	up	GO:0032040 (cellular_component:small-subunit processome); GO:0031428 (cellular_component:box C/D snoRNP complex); GO:0005730 (cellular_component:nucleolus);	map03008 (Ribosome biogenesis in eukaryotes)

								GO:0000272 (biological_process:polysaccharide catabolic process); GO:0005576 (cellular_component:extracellular region); GO:0008061 (molecular_function:chitin binding); GO:0004553 (molecular_function:hydrolase activity, hydrolyzing O-glycosyl compounds); GO:0004568 (molecular_function:chitinase activity); GO:0030515 (molecular_function:snoRNA binding); GO:0006032 (biological_process:chitin catabolic process)	
LYS21	TRINITY_DN934_c0_g1	homocitrate synthase lys21 [Fusarium torreyae]	4.224395	2.078745	6.99E-05	0.000368	up	GO:0019878 (biological_process:lysine biosynthetic process via aminoadipic acid); GO:0004410 (molecular_function:homocitrate synthase activity); GO:0005739 (cellular_component:mitochondrion)	map00300 (Lysine biosynthesis); map00620 (Pyruvate metabolism)
pckA	TRINITY_DN466_c0_g1	Phosphoenolpyruvate carboxykinase (ATP	2.59852	1.37769	4.89E-10	7.09E-09	up	GO:0016310 (biological_process:phosphorylation); GO:0005829 (cellular_component:cytosol); GO:0016301 (molecular_function:kinase activity); GO:0004612 (molecular_function:phosphoenolpyruvate carboxykinase (ATP) activity); GO:0005524 (molecular_function:ATP binding); GO:0006094 (biological_process:gluconeogenesis)	map00020 (Citrate cycle (TCA cycle)); map00010 (Glycolysis / Gluconeogenesis); map00620 (Pyruvate metabolism); map00710 (Carbon fixation by Calvin cycle)
UTP22	TRINITY_DN3667_c0_g1	U3 snoRNP protein [Fusarium torreyae]	3.131857	1.647018	0.000902	0.00352	up	GO:0032040 (cellular_component:small-subunit processome); GO:0034456 (cellular_component:UTP-C complex); GO:0006409	map03008 (Ribosome biogenesis in eukaryotes)

								(biological_process:tRNA export from nucleus); GO:0003723 (molecular_function:RNA binding); GO:0032545 (cellular_component:CURI complex); GO:0006364 (biological_process:rRNA processing)	
LYS4	TRINITY_DN2087_c0_g1	mitochondrial Homoaconitase [Fusarium torreyae]	7.15958	2.839875	3.79E-11	6.46E-10	up	GO:0019878 (biological_process:lysine biosynthetic process via aminoadipic acid); GO:0046872 (molecular_function:metal ion binding); GO:0051539 (molecular_function:4 iron, 4 sulfur cluster binding); GO:0005739 (cellular_component:mitochondrion); GO:0004409 (molecular_function:homoaconitate hydratase activity)	GO:0019878 (biological_process:lysine biosynthetic process via aminoadipic acid); GO:0046872 (molecular_function:metal ion binding); GO:0051539 (molecular_function:4 iron, 4 sulfur cluster binding); GO:0005739 (cellular_component:mitochondrion); GO:0004409 (molecular_function:homoaconitate hydratase activity)
LYS2	TRINITY_DN10798_c0_g1	large subunit of alpha-aminoadipate reductase [Fusarium torreyae]	4.527273	2.178642	7.08E-11	1.15E-09	up	GO:0009085 (biological_process:lysine biosynthetic process); GO:0044550 (biological_process:secondary metabolite biosynthetic process); GO:0004043 (molecular_function:L-aminoadipate-semialdehyde dehydrogenase activity); GO:0031177 (molecular_function:phosphopantetheine binding); GO:0019878 (biological_process:lysine biosynthetic process via aminoadipic acid)	GO:0009085 (biological_process:lysine biosynthetic process); GO:0044550 (biological_process:secondary metabolite biosynthetic process); GO:0004043 (molecular_function:L-aminoadipate-semialdehyde dehydrogenase activity); GO:0031177 (molecular_function:phosphopantetheine binding); GO:0019878 (biological_process:lysine biosynthetic process via aminoadipic acid)
fbp	TRINITY_DN3053_c0_g1	Fructose-1,6-bisphosphatase [Fusarium torreyae]	2.813396	1.492313	9.72E-06	6.21E-05	up	GO:0042132 (molecular_function:fructose 1,6-bisphosphate 1-phosphatase activity); GO:0005829	GO:0042132 (molecular_function:fructose 1,6-bisphosphate 1-phosphatase activity);

								(cellular_component:cytosol); GO:0030388 (biological_process:fructose 1,6-bisphosphate metabolic process); GO:0005986 (biological_process:sucrose biosynthetic process); GO:0046872 (molecular_function:metal ion binding); GO:0006000 (biological_process:fructose metabolic process); GO:0006002 (biological_process:fructose 6-phosphate metabolic process); GO:0006094 (biological_process:gluconeogenesis)	GO:0005829 (cellular_component:cytosol); GO:0030388 (biological_process:fructose 1,6-bisphosphate metabolic process); GO:0005986 (biological_process:sucrose biosynthetic process); GO:0046872 (molecular_function:metal ion binding); GO:0006000 (biological_process:fructose metabolic process); GO:0006002 (biological_process:fructose 6-phosphate metabolic process); GO:0006094 (biological_process:gluconeogenesis)

Note:

gene_id: Unique gene identifier. A distinct ID assigned to each transcript or dereplicated unigene by the analysis pipeline for tracking and identification.

nr_descrip: Functional description based on the NCBI non-redundant protein database. Provides the most probable biological function annotation for the gene product.

fc: Fold change. Typically the ratio of expression level in the experimental group to that in the control group, directly reflecting the magnitude of expression change. fc > 1 indicates up-regulation, fc < 1 indicates down-regulation.

log2fc: Logarithm (base 2) of the fold change. A positive value indicates up-regulation, a negative value indicates down-regulation. This metric is commonly used in subsequent statistical analyses and visualizations.

pvalue: Raw P-value from the statistical test for differential expression, used to assess if the difference is likely due to sampling error. Typically, $P < 0.05$ is considered statistically significant.

padjust: Adjusted P-value. Obtained after correction for multiple hypothesis testing, used to control the false discovery rate. padjust < 0.05 is generally considered indicative of significant differential expression.

regulate: Direction of expression regulation. Indicates whether the gene is up-regulated or down-regulated in the experimental group compared to the control.

go: Gene Ontology functional classification ID. Denotes the biological processes, cellular components, or molecular functions the gene is involved in.

paths: KEGG pathway map identifiers. Lists the identifier(s) of one or more KEGG pathways the gene participates in.

Table S16 List of differentially expressed metabolites identified in the metabolomic analysis

Metabolite	P_value	FDR	FC (GE/CK)	Log2FC (GE/CK)	Regulate	KEGG Pathway Description	Library ID	HMDB Superclass	CAS ID	M/Z	Adducts	Formula
Ginkgolic Acid	1.75E-12	2.54E-10	35.6473	5.1557	up	-	HMDB0033897	Benzenoids	22910-60-7	345.2434	M-H	C22H34O3
postin	5.64E-18	6.04E-15	6.1749	2.6264	up	-	HMDB0005772	Organic acids and derivatives	103745-46-6	519.3046	M+Na	C22H40N8O5
Vitamin P	1.10E-21	8.27E-18	3.5774	1.8389	up	Metabolic pathways; Biosynthesis of secondary metabolites	HMDB0003249	Phenylpropanoids and polyketides	153-18-4	609.1462	M-H	C27H30O16
Isopersin	2.11E-13	4.33E-11	3.5343	1.8214	up	-	HMDB0032735	Lipids and lipid-like molecules	–;	361.2749	M-H2O-H	C23H40O4
(3R, 6'Z)-3,4-Dihydro-8-Hydroxy-3-(6-Pentadecenyl)-1H-2-Benzopyran-1-One	1.98E-15	7.08E-13	3.3021	1.7234	up	-	HMDB0041301	Organoheterocyclic compounds	158627-94-2	371.259	M-H	C24H36O3
Kaempferol-3-O-(6'''-Trans-P-Coumaroyl-2''-Glucosyl)Rhamnoside	3.69E-20	1.38E-16	2.537	1.3431	up	-	-	-	-	739.1883	M-H	C36H36O17
Ginkgolide C	1.40E-12	2.09E-10	2.9421	1.5568	up	-	HMDB0036860; LMPR0104540003	Lipids and lipid-like molecules	15291-76-6;	439.1247	M-H	C20H24O11
Xenognosin B	0.000132	0.000556	3.7139	1.8929	up	Metabolic pathways; Biosynthesis of secondary metabolites	HMDB0031720	Phenylpropanoids and polyketides	1890-99-9;	567.1289	2M-H	C16H12O5
2,3,4-Trihydroxybenzoic Acid	1.81E-13	3.82E-11	3.6862	1.8821	up	-	HMDB0059964	Benzenoids	610-02-6	169.0143	M-H	C7H6O5
Maesopsin	1.04E-19	2.73E-16	7.0659	2.8209	up	Metabolic pathways	-;LMPK12130072	-	5989-16-2;	287.056	M-H	C15H12O6
Docosahexaenoic Acid	4.53E-08	8.44E-07	2.418	1.2738	up	Biosynthesis of unsaturated	HMDB0002183	Lipids and lipid-like molecules	-;6217-54-5	387.2541	M+Hac-H	C22H32O2

						fatty acids						
Kaempferol 3-O-Robinoside	4.55E-11	3.79E-09	2.3198	1.214	up	-	-	-	-	593.1514	M-H	C27H30O15
1,28-Octacosanediol Diferulate	2.75E-16	1.25E-13	2.586	1.3707	up	-	HMDB0038065	Phenylpropanoids and polyketides	-	777.5309	M-H	C48H74O8
Ibuprofen Glucuronide	6.27E-18	6.26E-15	10.4448	3.3847	up	Metabolic pathways; Pentose and glucuronate interconversions; Ascorbate and aldarate metabolism; Biosynthesis of cofactors	HMDB0010343	Organic oxygen compounds	98649-76-4;_	427.1613	M+FA-H	C19H26O8
Ginkgolide B	4.41E-13	8.07E-11	2.2372	1.1617	up	-	HMDB0036861; LMPR0104540002	Lipids and lipid-like molecules	15291-77-7;	423.1297	M-H	C20H24O10
St(24:2_O4)	1.09E-19	2.73E-16	2.1998	1.1374	up	-	-	-	-	389.27	M-H	C24H38O4
2-Hydroxy-4-Pentadecylbenzoic Acid	1.11E-12	1.73E-10	2.1799	1.1242	up	-	-	-	-	347.259	M-H	C22H36O3
12B-Hydroxy-5B-Cholanoic Acid	8.99E-21	4.49E-17	3.1002	1.6324	up	-	HMDB0002431	Lipids and lipid-like molecules	15173-23-6	397.2747	M+Na-2H	C24H40O3
5-(12-Heptadecenyl)-1,3-Benzenediol	3.76E-12	4.66E-10	2.1529	1.1063	up	-	HMDB0038527	Benzenoids	103462-06-2	345.2801	M-H	C23H38O2
Diacetoxyscirpenol	2.49E-10	1.51E-08	3.0455	1.6067	up	-	HMDB0035104	Lipids and lipid-like molecules	2270-40-8	411.1661	M+FA-H	C19H26O7
D8'-Merulinic Acid C	4.19E-08	7.92E-07	1.6915	0.7583	up	-	HMDB0041453	Benzenoids	69506-63-4	373.2747	M-H	C24H38O3
Petrosaspongiolide M	5.34E-11	4.28E-09	1.8111	0.8569	up	-	HMDB0256357	Lipids and lipid-like molecules	-	441.2623	M-H2O-H	C27H40O6
Rutarin	3.59E-10	1.95E-08	5.9403	2.5705	up	-	HMDB0030884	Phenylpropanoids and polyketides	20320-81-4	423.1299	M-H	C20H24O10
Allylestrenol	6.33E-15	1.94E-12	1.9866	0.9903	up	-	HMDB0015500	Lipids and lipid-like molecules	432-60-0;	359.2591	M+Hac-H	C21H32O
20, 22-Dihydrodigoxigenin	1.66E-13	3.56E-11	2.5412	1.3455	up	-	HMDB0060730	Lipids and lipid-like molecules	-	413.2312	M+Na-2H	C23H36O5
Luteolin	1.02E-16	5.65E-14	2.4054	1.2663	up	Metabolic pathways;	HMDB0005800; LMPK12110006	Phenylpropanoids and polyketides	491-70-3;	285.0405	M-H	C15H10O6

						Biosynthesis of secondary metabolites						
17-Beta-Estradiol 3-Sulfate-17-(Beta-D-Glucuronide)	6.68E-11	5.11E-09	1.7096	0.7737	up	-	HMDB0010358	Lipids and lipid-like molecules	-	567.1272	M+K	C24H32O11S
Apigenin	6.55E-09	1.79E-07	2.2335	1.1593	up	Metabolic pathways; Biosynthesis of secondary metabolites	HMDB0002124; LMPK12110005	Phenylpropanoids and polyketides	520-36-5;	269.0456	M-H	C15H10O5
Marmesin Galactoside	2.78E-06	2.54E-05	2.4483	1.2918	up	-	HMDB0041508	Phenylpropanoids and polyketides	156363-69-8	407.1349	M-H	C20H24O9

Note: List of differentially expressed metabolites identified in the non-targeted metabolomic analysis comparing GE-treated and control groups. The table provides the metabolite name, statistical significance (p-value, FDR-adjusted p-value), fold-change (FC and log_2FC), regulation direction, KEGG pathway annotation, and key identification details (HMDB ID, superclass, CAS number, measured m/z, adduct ion, and molecular formula). The "KEGG Pathway Description" field is marked as "-", indicating that a specific pathway assignment was not available in the queried database at the time of analysis.
